# The Influence of Roughness on the Properties of Electroactive Polypyrrole

**DOI:** 10.3390/molecules29225436

**Published:** 2024-11-18

**Authors:** Sylwia Golba, Julian Kubisztal

**Affiliations:** Institute of Materials Engineering, University of Silesia, 75 Pulku Piechoty Street 1A, 41-500 Chorzow, Poland; julian.kubisztal@us.edu.pl

**Keywords:** polypyrrole, conductive polymeric material, roughness, wettability, electrodeposition

## Abstract

This study describes the properties of electroactive polypyrrole and its applications, with a focus on the roughness of the material. This parameter is crucial as it influences the applicability of coated layers, leading to highly adherent coatings or programmed wettability. The first raised aspect covers the electrodeposition procedure, which can help tailor the desired smoothness determined by roughness parameters. Features such as the deposition method, synthetic solution components, potential boundaries, substrate type, and utilized additives are evaluated. In the following section, the application aspects are discussed with a focus on modern, currently developed subjects such as medical applications, including cell-adherent coatings, antibacterial coatings, and drug delivery modules, as well as more technological fields like improved adhesion to the substrate and the improved mechanical properties of the deposited coating.

## 1. Introduction

Polypyrrole (PPy) is an organic electroactive polymer (EAP) applicable in multiple fields, including coating layers [1,2], sensors [3], drug delivery modules [4], charge storage materials [5], and photosensitive elements for anticancer therapy [6]. Its usability is relevant to the electronic structure and topographical features that can be tuned by the synthesis conditions. The polymer can be synthesized using several techniques, including the chemical oxidative process [7] and electrochemical oxidation [8]. There are also some less commonly used techniques, such as the radiolytic technique [9], sono-supported process [10], and cell-assisted enzymatic process [11]. Studies of the electropolymerization mechanism prove that the process starts with monomer oxidation, followed by the coupling reaction leading to chain length extension [12,13]. Both the electron-transfer reaction and mass-transport process influence the redox activity of PPy, leading to structural changes in the form of conformation changes, swelling, shrinking, or relaxation [14].

The substrate used for electrodeposition (ED) is dictated by the materials’ prospective application and plays the role of a functional working electrode in electrosynthesis. Conveniently, it is frequently composed of materials stable at anodic potentials, like Pt [15] or ITO-coated glasses (In_2_O_3_/SnO_2_) [16]. Furthermore, other substrates were tested, including stainless steel [17], iron [18], copper [19], nickel [20], titanium [21], NiTi alloy [22], tantalum [23], glassy carbon [24], graphite [25], aluminum [8], and tungsten [26]. However, oxidative conditions may lead to parallel active metal dissolution; hence, a protective adlayer should be added to prevent this process [27]. In each case, the pyrrole monomer must distribute and diffuse on the surface of the substrate, be physically adsorbed, form an oxidized film, and then start to nucleate and grow, resembling the curvature or roughness of the underlying surface and, therefore, mirroring its shape [28]. Charged polymer attracts ions to balance the charge; hence, counterions are incorporated into the matrix due to electrostatic interactions [13]. In the subsequent step, electroneutrality is restored by either the expulsion of the anions or by the incorporation of cations from the electrolyte solution.

PPy is a polymeric material with the optical and electrical properties of (semi)metals [29]. Its conduction mechanism is strongly related to the movement of charge carriers formed along the conjugated chain (polarons and bipolarons (biradical cations)) [13]. Usually, for small dopant ions, one anion interacts with 3–4 pyrrole units, accounting for 30–40% of the weight of the polymer [30]. Different ions were used to dope PPy, including simple inorganic-like chloride [31], large organic-like dodecylbenzene sulfonate DBS^−^ [32], organic-like dodecyl sulfate DS^−^ [33], or polymeric type-like polystyrene sulfonate (PSS^−^) [34]. Depending on the size of the dopant ion (small-, medium-, and large-sized anions), PPy acts as an anion-, anion/cation-, and cation-exchange material, respectively.

Most of the available synthetic procedures, substrates, concentration regimes, additives, and dopants lead to final materials with various properties. Polypyrrole includes materials of various morphologies, agglomeration stages, crystallinity degrees, doping degrees, and charge-storage capacities. Hence, in this work, we focus on one of the urgent parameters from the application point of view, which is roughness. This property is a factor that influences cell behavior, like topography [35] and Young’s modulus [36]. We provide a broad scope of information on how the synthesis conditions influence the forming layer roughness and also how the roughness modifies the layer’s properties, including adsorption properties and wettability.

## 2. Roughness of the Coating

**Figure 1:**
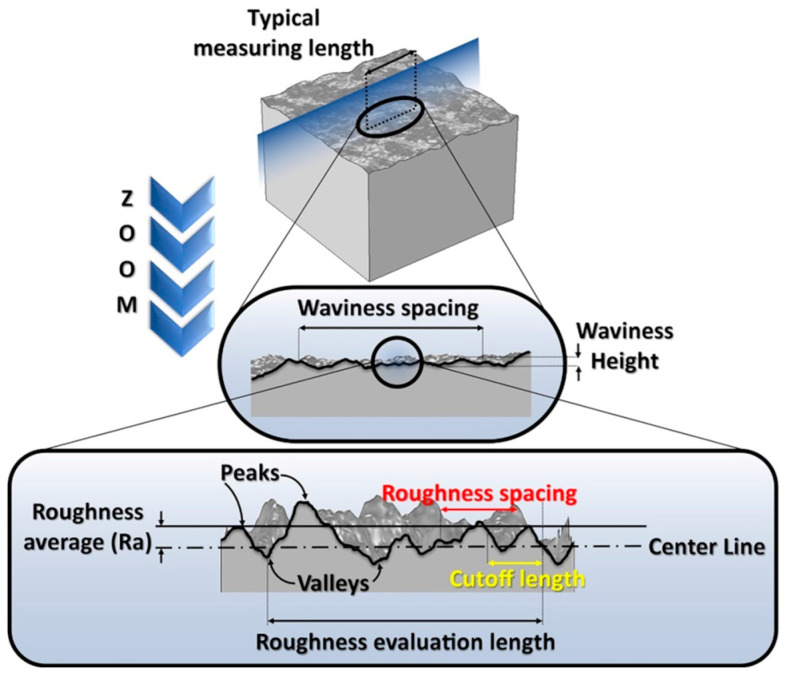
Schematic representation of the surface topography (adapted from [37] under a license agreement).

A schematic representation of surface topography is shown in Figure 1. Components of surface topography, such as roughness, waviness, and texture, can be modified via various surface modifications and machining methods [37]. The roughness of the surface is a feature that can particularly affect the behavior of polymers and, consequently, their applicability [38]. A rough surface provides anchoring sites for film deposition and molecule adsorption, playing a significant role in effective biofunctionalization to achieve antibacterial and neuroprotective effects [39], surface modification of neural-recording electrodes [40], and protein adsorption [41]. Today, both two-dimensional (2D) and three-dimensional (3D) methods of surface imaging are available. Depending on the technique used, profile (2D) and areal (3D) parameters can be determined based on experimentally registered data. Many parameters quantitatively describe surface features, including amplitude (height), spatial, hybrid, functional, volume, fractal, peak-to-valley, and motif parameters. A comprehensive description of both profile and areal parameters can be found in the ISO 21920 [42] and ISO 25178 standards [43] (ISO: International Organization for Standardization, www.iso.org).

Profile parameters are divided into three groups depending on the type of profile from which they are calculated, i.e., primary profile (P), roughness profile (R), or waviness profile (W) (see Figure 1). In practice, the parameters most commonly used to describe the surface topography of PPy are the arithmetic average of the roughness profile (*R_a_*) and the root-mean-square average of the roughness profile (*R_q_*). These parameters are determined using the following equations:

(1) $$R_{a}=\frac{1}{N}\sum_{i=1}^{N}\left|Z_{i}-\bar{Z}\right|$$

(2) $$R_{q}=RMS={\left\lceil \frac{1}{N}\sum_{i=1}^{N}{\left(Z_{i}-\bar{Z}\right)}^{2}\right\rceil }^{\frac{1}{2}}$$

where *N* is the number of data points, *Z_i_* represents the distance of the *i*-th point from the mean level, and $\bar{Z}$ denotes the mean value of the heights. Equations (1) and (2) show that the mean roughness (*R_a_*) is similar to the *R_q_* value, with the difference being that *R_a_* is calculated from the sum of absolute deviations from the mean, while *R_q_* is based on the sum of squared deviations. Additionally, it is important to note that Equation (2) corresponds to the standard deviation of the height distribution.

Profile parameters R_a_ and R_q_ can be extended to equivalent areal parameters S_a_ and S_q_:

(3) $$S_{a}=\frac{1}{\left(MN\right)}\sum_{j=1}^{M}\sum_{i=1}^{N}\left|Z_{i}-\bar{Z}\right|$$

(4) $$S_{q}={\left\lceil \frac{1}{\left(MN\right)}\sum_{j=1}^{M}\sum_{i=1}^{N}{\left(Z_{i}-\bar{Z}\right)}^{2}\right\rceil }^{\frac{1}{2}}$$

where *N* is the number of points per profile, and *M* is the number of profiles. The values of *S_a_* and *S_q_* parameters depend on height deviations from the mean surface level. In contrast to the profile parameters *R_a_* and *R_q_*, *S_a_* and *S_q_* can represent primary, rough, or wavy surface features, depending on the pre-filtering methods applied before calculating the parameters.

## 3. Tools Used to Measure Roughness of the Coating

Several methods are commonly used for roughness determination, including profilometry, atomic force microscopy, and optical and ultrasonic techniques. Each method employs a distinct measurement principle: contact profilometry uses a stylus tip that physically touches the surface and moves across it [44]; AFM involves a cantilever with a sharp, pyramidal or tetrahedral tip that scans the surface at the atomic level in contact, non-contact, or tapping mode [45]; and optical [46] or ultrasonic techniques [47] use electromagnetic or sound waves to probe the surface of materials. The majority of the analyses are conducted with profilometry or AFM imaging. AFM is one of the techniques most widely used to characterize surface topography and simultaneously measure and map the elastic properties of polymer surfaces [48], even under liquid-environment conditions. It measures molecular forces with a sensitivity down to the range of 1–10 pN [49], which assists in probing mechanical properties at the nano-micro level [50]. The technique has been used to investigate the actuation of films on redox stimulation [51], while AFM analysis of films under liquids has been used to provide data relevant to in vivo performance conditions.

An AFM image is a simulated image based on the height of each surface point, with each point of the surface prescribed to a height. According to the height distribution and height difference between different areas of the surface, accurate information about surface roughness can be calculated [52]. Specifically for conducting polymers (CPs), the electrochemical surface properties may be characterized at the nanoscale level during electrical stimulation via electrochemical atomic force microscopy (EC-AFM). EC-AFM studies on films grown on a gold Mylar working electrode revealed dynamic roughness or height changes as a function of the electrical stimulus [34]. Moreover, morphology changes associated with the doping/dedoping process have revealed a distinct reorganization of the film that is detectable by the technique. Profilometric analysis is a technique used to quantify the morphology of material surfaces by measuring the peaks and the valleys on the surface. Several technologies are available, including contact profilometry and optical profilometers [53]. The experimentally generated data can be used to create two-dimensional (2D) and three-dimensional (3D) surface profile graphs of the object [54]. The resolution of AFM exceeds the level of accuracy of a traditional profilometer [55], but the AFM’s broadest measurement range is restricted to a surface of 100 μm × 100 μm [56]. Hence, the results derived from both techniques complement each other, delivering a broad and detailed description of the material.

## 4. Roughness Evolution at the Synthesis Stage

**Figure 2:**
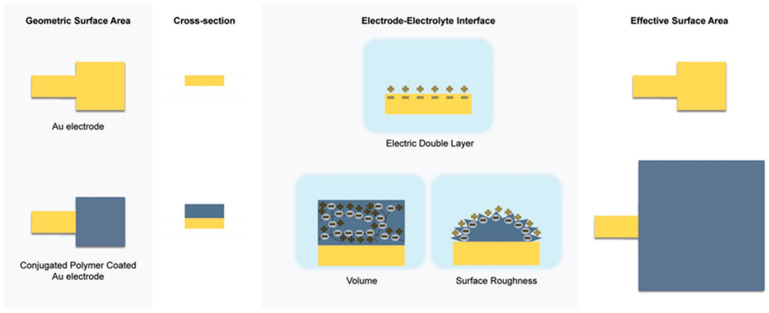
The increase in the effective surface area of a metal electrode coated with CPs (adapted from [58] under a license agreement).

The yield and quality of the product deposited in electrosynthesis are determined by multiple physicochemical factors, including pH; electrode nature (e.g., the material, shape, porosity); temperature; nature and concentration of the monomer/counterion; applied procedure; and potential/current density value, solvent nucleophilicity, and stirring conditions [12]. The type and concentration of the dopant employed in the film deposition process alter the morphology, roughness, wettability, and surface charge, allowing its functionalization, e.g., for specific biomedical applications [57]. The coating of the substrate surface with CP increases the effective surface area of the electrode, which is schematically pictured in Figure 2. CP film thickness is typically in the range of nm to µm, and porosity and surface roughness are about nm to µm [58]. It was estimated that covering the surface with CP extends the surface area 40 times.

The deposition of PPy film on the electrode proceeds via the mechanism proposed by several groups [27,59]. One of the stages is the formation of the oligomeric species, which leads to the formation of the so-called oligomer density region (ODR) in the vicinity of the electrode. The surface roughness is governed by the polymer-nucleation and deposition-growth mechanisms [59], with the nucleation stage depending largely on the substrate character. The investigation of the stages of the formation of a thin PPy layer on an atomically flat Au (111) surface [60] revealed that the thin film deposited via the polymerization of previously adsorbed monomer species.

**Figure 3:**
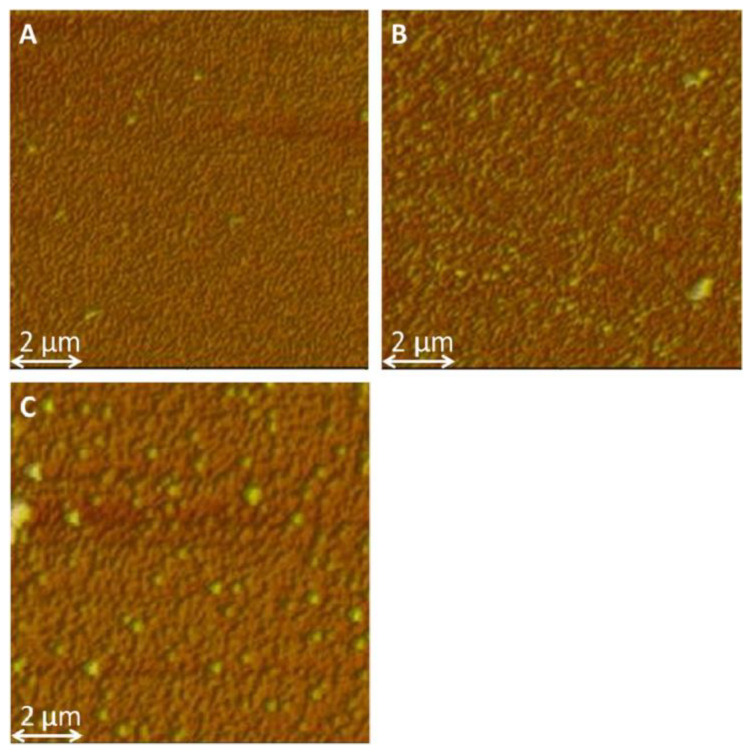
AFM images of the surface topography of PPy films deposited on a gold electrode: (**A**) potentiostatically, (**B**) potentiodynamically, (**C**) galvanostatically (adapted from [69] under a license agreement).

The external signal applied to oxidize the monomer can have different forms; hence, several electropolymerization techniques may be used, including potentiostatic [61], galvanostatic [62], potentiodynamic [33], or pulsed ones [63]. During the deposition process, careful steps should be taken to avoid the overoxidation process [64,65], as severe changes in material properties may occur after being exposed to oxidizing conditions or very positive electrode potentials [64]. CP film thickness, as well as its porosity and surface roughness, vary depending on processing methods [58]. Faster electrodeposition is observed for the galvanostatic technique at a high current density or potentiostatic one at a high electrode potential; still, it leads to the formation of rougher surfaces [66], resulting in inferior properties [67]. The surface roughness is also governed by polymer-nucleation (instantaneous or progressive) and deposition-growth mechanisms (two-dimensional or three-dimensional), as shown for glassy carbon [59] and platinum [68] working electrodes. The influences of the electrochemical deposition method on the internal film structure and solvation were discussed by Hillman et al. [69]. A decreasing polymer segment density and increasing porosity of the samples were reported for the samples deposited with the following methods: potentiostatic (PS) < potentiodynamic (PD) < galvanostatic (GS). The exterior surfaces of the growing films were rough, most pronounced under galvanostatic control, as evidenced by AFM imaging (Figure 3). The initial solvent content was greater in the GS-deposited film, which may result in external roughening of the film during drying and/or internal stresses that trigger delamination. Smoother films were produced by potentiodynamic polymerization [70] as more nucleation sites were formed, reducing the 3D growth of macrochains. For the 2D mechanism, a layer-by-layer deposition process prevailed, which formed a more compact product, while the continuous growth of nuclei in 3D produced porous films [33,59].

For polypyrrole on Mg-Al alloy (AZ91D) electrodes, higher initial and end potential values in electropolymerization increased the coating roughness and irregular surface morphologies. This transferred to worse adhesion behavior after immersion in Na_2_SO_4_ solution detected in the adhesion tests [71]. The authors determined the relationship between adhesion, conductivity, and corrosion resistance prescribed for PPy overoxidation. Controlling the magnitude of the applied electrical field induced some effects on the topography of the freshly formed polymer. PPy films prepared at lower anodic potentials were denser and more homogeneous [71]. The electrochemical polymerization of pyrrole supported by high-frequency sonication on Si substrates led to coatings with a smaller roughness than non-sonicated ones [72] with thinner but more homogeneous surface structures. Still, the adhesion forces between sonicated polypyrrole films and Si substrates were very weak [72].

Morphological parameters, including the roughness of PPy electrochemically deposited on vitreous carbon substrates as a function of the nature of the dopant, were investigated by J. G. Ibanez [73]. Based on the electrochemical data, thicker films were deposited for the constant potential (CP) procedure than for the cyclic voltammetry (CV) one. In addition, the film thickness was higher in more acidic conditions, with the applied potential as a key factor that governs the final properties of the film. The roughness expressed as the R_q_ was 3.62 nm for the pure vitreous carbon substrate, while for the deposited PPy films, the values increased along with film thickness growth in the order of approx. 72 nm (for H_2_SO_4_), 42 nm (for K_2_SO_4_), 38 nm (for HCl), and 21 nm (for KCl) [73]. The result showed that the surface roughness and topographic features can be adjusted with the choice of the pH value and electrodeposition parameters. A similar analysis presented in [74] focused on a group of inorganic doping anions, namely, I^−^, Br^−^, Cl^−^, F^−^, NO_3_^−^, ClO_4_^−^, and SO_4_^2−^. Film thickness increased with the size of the anion in halide electrolytes, while larger anions provided more uniform structures, as concluded based on R_q_ values [74]. Surface topography described by the R_a_ parameter of PPy electrodeposited on 0.1 mm thick Pt foil was studied by Compton [75,76]. Several thicknesses and anion-doped (chloride, sulfate, perchlorate, and dodecyl sulfate) films were studied using atomic force microscopic imaging and the fractal dimension concept. The surface morphology of the thin film (<1 µm) was less affected by the dopant type, where globular-shaped polymer particles were mainly oriented along scratch lines of the substrate. Differences were found for thick layers (>1 µm) with two types of globules of various heights present on the film surface. This was converted into a variation of R_a_ parameter values.

Organic anions of various origins are interesting, as their presence in the material’s structure can impart a new functionality [77]. A comparison of the film roughness (deposited on a gold-coated Mylar substrate) for several organic anion-doped PPys was reported by Wallace [78]. The differences in the dimensions of the nodular structures were taken as indicators of changes in the roughness of the coating. The incorporation of large hyaluronic acid (HA) and p-toluenesulfonic acid (pTS) led to films with the roughest surface R_a_ values of 32.3 nm and 30.1 nm, followed by dextran sulfate (DS) (25.2 nm), while chondroitin sulfate A (CS) and poly(2-methoxyaniline-5-sulfonic acid) (PMAS)-doped films were markedly smoother (12.1 nm and 5.8 nm). The topographical uniformity was estimated as non-uniform, comprising large nodule structures with smoother areas for HA and pTS systems. At the same time, for DS, CS, and PMAS ones, the topographical uniformity was assessed as more uniform with nodular organization [78]. Interestingly, as PPy and PMAS were redox active, it led to mutual switching abilities of a PPy/PMAS composite as discussed by Zhao [79]. The roughness measurements shown for electrodeposited PPy films on FTO (fluorine-doped tin oxide) [66] further indicate that the salt nature significantly affects roughness parameters. Large anions (e.g., p-toluenesulfonate (TSO^−^) or 1-naphthalenesulfonate (NS^−^)) led to small values of roughness parameters, confirming that the polymer samples were flat and homogeneous. On the contrary, polymer films containing small anions (perchlorate (ClO_4_^−^), nitrate (NO_3_^−^), and tetrafluoroborate (BF_4_^−^)) led to coatings with higher roughness values [66]. The influence of the surrounding medium on the PPy film roughness was presented for perchlorate-doped polypyrrole thin film (<100 nm) [48]. The roughness changed from 2.3 nm to 2.8 nm upon immersion of the film in the electrolyte, with a swelling ratio of up to 17%·V^−1^ and changes in elastic modulus of up to 80%, accompanied by potential independent film swelling attributed to osmotic expansion [48]. The relationships of polypyrrole film thickness and Young modulus versus the electrical potential follow nearly linearly, increasing due to solvent and ion influx as the film is oxidized and decreasing during reduction. The surface topography of polypyrrole films synthesized galvanostatically on a stainless steel electrode by Kaynak [80] was mainly governed by the organic dopant concentration (p-toluenesulfonate anion), synthesis time, and subsequent heat treatment. The arithmetic average of the roughness profile (R_a_), the root mean square average of the roughness profile (R_q_), and the nodule diameter increased with the increasing dopant concentration and synthesis time. A smoother surface morphology with low R_a_ values of appr. 50.0 nm was reported for lightly doped films, while dendritic surfaces with R_a_ values of the nodules of several microns were characterized for highly doped ones. Extended synthesis times led to the transformation of the surface topography from typical cauliflowers into a worm-like fibrillar structure [80]. Polypyrrole doped with polystyrene sulfonate [34] grown galvanostatically (a current density of 1.3 mA·cm^−2^) on a gold-coated Mylar substrate as a working electrode was studied as the prototype of CP-based actuators. Various growth times resulted in obtaining different thicknesses and influenced film roughness—an extended time provided significantly rougher surface morphology. The thickness vs. polymerization charge dependence is linear, with a rate of 3.91 μm·C^−1^·cm^2^ [34]. Electrochemically deposited PPy and poly(methylene blue) (PMB) copolymer on ITO-coated glass slides was traced by Ramanaviciene [81]. Three saccharides (namely, lactose, sucrose, and heparin) were used as dopants in the CV process. The surface of the Lac-doped layer was most evenly distributed, while the most massive surface structures were formed on the Hep-doped layer. The roughness increased in the order of Lac-, Suc-, and Hep-doped systems, with a maximum peak height of 1.5–2.5 μm, 2 μm, and 3 μm. The layers tended to wrinkle along with the drying process after the synthesis stage. Interestingly, all three layers showed electrochromic behavior, while the Hep-doped coating was the most mechanically stable. Pyrrole electrochemical polymerization in the presence of salt and acid forms of flexible-chain sulfoacid polyelectrolytes was reported by Gibkova [82]. Synthesis in the presence of polyelectrolytes in acid form occurred faster for deposition in potentiostatic, galvanostatic, or potentiodynamic modes onto a SnO_2_ glass electrode. The surface topography was typical, with regular globular structures. As a result, the roughness of the acid–polyelectrolyte-doped films was slightly higher (R_a_ 18.4–24.6 nm) than that deposited from salt-form solutions (R_a_ 17.7–19.3 nm). Thicker films were prepared in the presence of polyelectrolytes (140 nm) than in inorganic electrolytes (80 nm).

**Figure 4:**
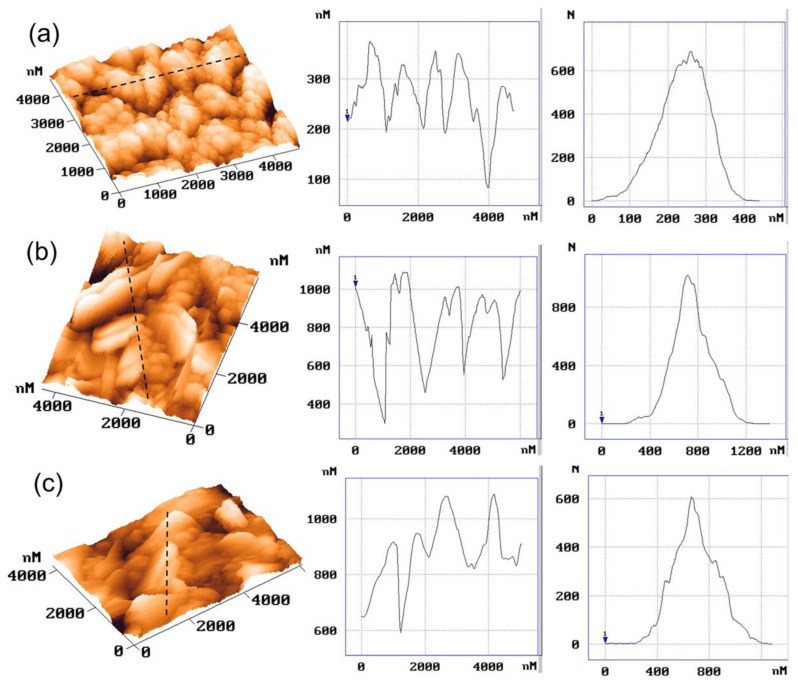
AFM images, sectional analysis, and height distribution diagrams for (**a**) PPy-oxalic (**a**), and (**b**,**c**) PPy-DS; the arrow with number refers to starting point of the measurement (adapted from [33] under a license agreement).

Some groups reported using surface active compounds as modifications utilized for ED. The PPy-DS (dodecyl sulfate ion) film on iron substrates showed the structure of thin multilayered plates, which was prescribed for preferential 2D growth [33]. The R_q_ parameter of the PPy-DS films was 160 nm, higher than that for the PPy-oxalic layer (65 nm) and resulting from the vertically oriented plates (Figure 4). The 2D structures were related to intense α-β coupling of pyrrole molecules, leading to highly crosslinked structures. Consequently, PPy-DS film exhibited greater protective properties than PPy-oxalic ones [33]. Conical polypyrrole was reported by Winkler [83] after electrochemical oxidative polymerization of Py in aqueous solutions containing NaClO_4_ and polymeric or anionic surfactants, namely, polyvinyl pyrrolidone (PVP) and SDS. Overall, the rate of polymer precipitation can significantly affect the structure of the forming polymer. The film originating from the NaClO_4_ solution was more irregular in surface morphology and rougher than the NaCl one. Material deposited potentiostatically on the Ti6Al7Nb alloy was obtained in the presence of various surfactants like poly(sodium 4-styrenesulfonate) (NaPSS), tert-octylphenoxy polyethoxyethanol (Triton X-100), or N-dodecyl-β-D-maltoside (DM) [84]. The presence of surfactants produced films of lower surface roughness compared to unmodified ones, while Triton X-100 additionally increased adhesion to the substrate.

**Figure 5:**
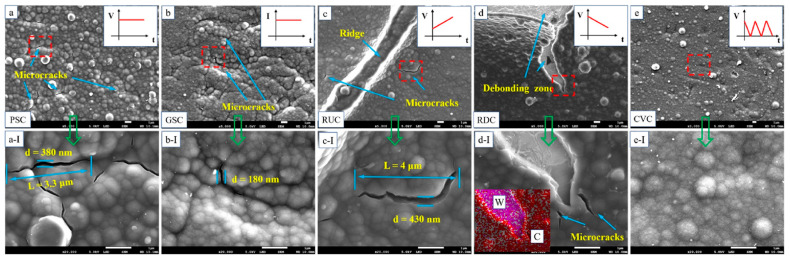
The surface morphology of different PPy–ZnO coatings after 500 million pulsing tests in a PBS solution, depositon with respective techniques: (**a**) potentiostatic, (**b**) galvanostatic, (**c**) ramp-up potential, (**d**) ramp-down potential, (**e**) cyclic voltammetry, figures of ((**a-I**–**e-I**)) serie represent magnification of the respective images of (**a**–**e**) serie in the area marked with red square The inserted image in d-I represents the result of EDS (Energy Dispersive X-Ray Spectroscopy) surface scanning with W for tungsten, C for carbon (adapted from [26] under a license agreement).

The ion-exchange properties of the matrix, along with the mobility of the counterion, are affected by the temperature and nature of the electrolyte solution in which the polymer is tested [85]. An analysis of the impact of synthetic protocols on morphology, including the fractal rough surface on perchlorate-doped PPy, was proposed by Mahjani [52]. An analysis of AFM images of polypyrrole electrodeposited on glassy carbon (GC) electrode revealed that synthesis in higher temperatures provides rougher films with a simultaneous larger surface area, resulting in increased electrochemical capacitance (from 45.2 to 88.1 µF·mm^−2^). An elevated temperature was also responsible for more intense oligomer formation, while lowering this temperature hampered monomer diffusion, slowing down the deposition process, which led to a smoother surface. Electrochemical tests (CV and electrochemical impedance spectroscopy (EIS)) confirmed this observation, providing higher electrochemical capacitance of films synthesized at higher temperatures. A similar observation was denoted by Alshammary [86], who reported an inverse relationship between the temperature and the surface roughness; as the temperature decreased, the film’s surface gradually increased, transferring to become more compact, coherent, and mechanically stronger. At the synthesis stage, higher temperatures increase the interaction between the molecules of the monomer, leading to film formation along with the activation of unwanted reactions (e.g., increasing the possibility of α-β and β-β coupling instead of a free polymer defect chain via desired α-α bonding). This was also true for the conjugation length, as longer conjugation of chains with a less defective structure was found for material deposited at lower temperatures. The effect of a prolonged deposition time on the surface roughness of PPy films was traced. It showed that the rougher surface morphology of the thicker films was correlated with higher-order fractal structures formed by the conglomeration of the 50–100 nm particles, as well as its enhanced porosity [34]. The presence of additives influences the growth direction and mechanism of deposition, as shown in studies by several groups. The electrochemical stability of a PPy-zinc oxide (PPy-ZnO) composite coating based on tungsten substrates was traced [26] by changing electrodeposition protocols. The coatings deposited using the potential governed techniques, like potentiostatic (PS, R_a_ = 203 nm), ramp-up potential (RU, R_a_ = 161 nm), and ramp-down potential (RD, R_a_ = 142 nm) methods, were formed with a higher deposition rate and coarser morphologies, which caused the generation of cracks. Using the galvanostatic (GS, R_a_ = 129 nm) and cyclic voltammetry (CV, R_a_ = 74 nm) methods led to a more compact structure and smoother morphologies obtained at lower deposition rates [26]. The surface morphology of coatings observed using SEM (Figure 5) showed microcracks formed by the repeated volumetric expansion and shrinking of coatings due to the absorption and extraction of counterions inside the microporous coatings [26].

The electrosynthesis and characterization of polypyrrole on stainless steel electrodes decorated with gold nanoparticles were described by Gervasi [17]. Films were grown in different electrolytes, leading to worm-like structures for perchlorate ions and columnar structures for salicylate-containing solutions. Cross-section profiles of the films showed that both R_a_ and R_q_ parameters increased when the electrolyte was changed from perchlorate to salicylate. Thus, an R_q_ of 0.78 μm was observed for perchlorate dopants characterized by the formation of relatively larger globular domains. In comparison, the R_q_ increased to 1.42 µm for salicylate dopants blamed for a columnar structure. The observed nanoscale roughness in the Au/PPy film structure was likely to facilitate charge transfer between pyrrole nitrogen and embedded gold nanoparticles (GNPs).

The composites prepared with either pristine or functionalized multi-walled carbon nanotubes (MWCNTs), oxidized (MWCNT-Ox), or pyrrole-modified (MWCNT-Py) were reported [87]. AFM images of PPy/MWCNT composites showed changes induced by nanotube insertion. A uniform, nano-pillar structure of pure PPy (R_q_ = 45 nm) inverted into regular domains of PPy-oriented chains induced by unmodified MWCNT [87] with the R_q_ = 80 nm. The functionalization of MWCNT raised the R_q_ parameters to a lesser extent (46 nm and 59 nm), which manifests much smoother layers. The incorporation of additives like polyethylene oxide (PEOx) in polypyrrole-doped dodecylbenzene sulfonate (PPy/DBS) linear actuators was proposed by Kiefer [88]. For composites produced galvanostatically on stainless steel working electrodes, electro-chemo-mechanical deformation measurements showed significant improvement in the electronic conductivity of the sample with 15% of PEOx accompanied by an enhanced strain compared to the pristine PPy/DBS film. The presence of the additive led to the formation of denser and more flexible films with smooth surfaces [88]. Optical profiling for the PPy-modified zinc surfaces [10] showed that, for all charge densities, ultrasound-supported synthesis led to surface refining. Films were deposited at a constant potential of 3.0 V vs. SCE for charge densities of 6 C·cm^−2^. The average surface roughness was reduced from 47% to 32% when using ultrasounds. A more compact and smoother surface resulted from the activation of the substrate surface via cavitation bubble collapse, which multiplied nuclei at the first stage of nucleation with the subsequent formation of smaller globules.

**Figure 6:**
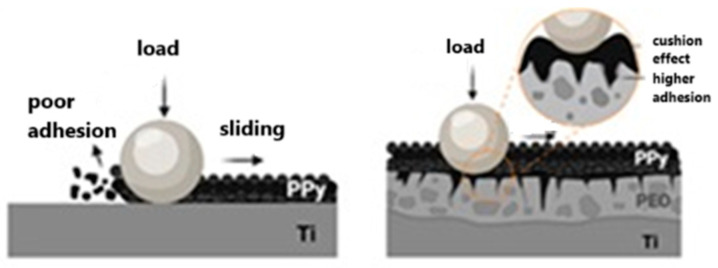
Some strategies used to improve the adhesion strength of the PPy film on metallic substrates: the wear process of PPy film during the tribological test for PPy-coated cpTi or PEO-modified substrate (adapted from [21] under a license agreement).

Some groups found post-synthesis modifications that can verify their impact on coating roughness. In the post-synthesis doping/dedoping cycles, PPy films become roughened and smooth with oxidation and reduction, respectively, as proved by cyclic voltammetry [89]. The electroactivity of PPy-coated iron was retained in a neutral solution when the system was scanned between −0.6 and 0.3 V. The increase in current experienced by the electrode for each new scan was associated with the roughening of the coated electrode surface. For the acidic solution, after the third cycle, the R_q_ parameter of iron increased from approximately 50 to 500 nm. The post-synthesis modification of doped polypyrrole coatings was also proposed by Siong Teh et al. [90]. The coating was synthesized in an anodic polymerization process and subsequently modified with externally applied potentials in an aqueous sodium dodecylbenzene sulfonate solution. The additional step led to roughening of the surface of doped polypyrrole, as manifested by the formation of a hierarchical micro- and nanoscale structure. The R_a_ parameter of the detected moieties, microscale islands, was transformed from 2.3 nm (at −0.8 V) to 14.6 nm (at +0.8 V). The procedure also influenced the final degrees of wetting, as static contact angles changed from 64° to 122° for the broad potential window (−0.9 to +0.9 V) [90]. Topographical and electronic properties of PPy were demonstrated by V. A. R. Barão [21] with an impact on the improvement in coating adhesiveness. As a tool for modification, the plasma electrolytic oxidation (PEO) technique was chosen, and commercially pure titanium (cpTi) discs were used in a step procedure (roughening and deposition). A highly adherent polymeric thin film was deposited on a PEO-modified substrate, as manifested in the wear process. The coating provided a lower friction coefficient and wear loss due to the cushion effect of the PPy film (Figure 6) [21].

## 5. The Impact of Roughness from Application Perspective

### 5.1. Improved Adhesion of Coating

**Figure 7:**
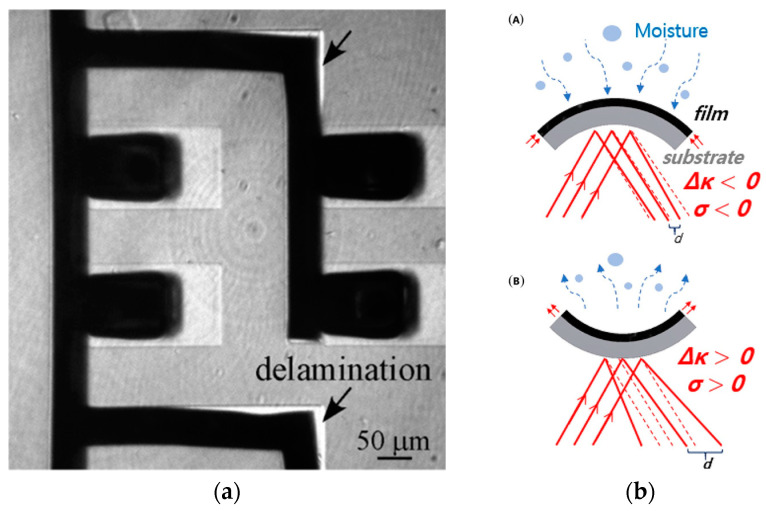
(**A**) The delamination of PPy from the underlying Au electrode (bent at an angle of approximately 20° from the surface) (reprinted with permission from [95]). Copyright: 2024 American Chemical Society; (**B**) schematic of the possible curvature and stress relationship due to moisture (**a**) absorption and (**b**) desorption (adapted from [96] under a license agreement).

The functionality of PPy coatings in practical applications depends on their adhesive properties. Surface roughness is important for adherent coatings, as a rougher surface delivers more sites to bind the coating on the surface. Still, a rough surface is prone to the corrosion process as it provides a less uniform structure, with cracks facilitating access to corrosive ions. Several paths can be used to improve it, like the incorporation of biomolecules, increasing the roughness of the nanostructured layers, providing hybrid layers, or changing the hydrophilicity of the surface. PPy is relatively stable in the oxidized form and slightly less in the reduced form. The mechanical properties, including the strength and adhesion of the coating materials to solid substrates, are vital for long-term applications [91,92]. Available reports validate the long-term performance of PPy actuators [93] and the long-term activity of bioelectronic interfaces [94]. Coating failure can be observed as delamination from the metal substrate and internal cracking. There is a need to optimize the deposition condition to ensure the durability of materials in long-term applications. In the operating mode, CP undergoes continuous switching between oxidized and reduced states [95]. This leads to expansion/contraction, which results in high shear stresses at the polymer/electrode interfaces. As a result, the polymer coating tends to delaminate, which reduces the efficiency of the device and slowly degrades its performance. Figure 7A shows a film of PPy delaminated from the Au electrode on the surface of a silicon wafer. The strong impact of moisture on delamination phenomena was proved and discussed by Sung Yeol Kim et al. [96], as its presence and transport provoke a change in curvature and stress (Figure 7B). Absorbed moisture enhances film expansion, leading to a negative curvature and compressive stress applied to the film. Moisture desorbing results in drying, which leads to film shrinking and hence a positive curvature with tensile stress applied to the film [96].

**Figure 8:**
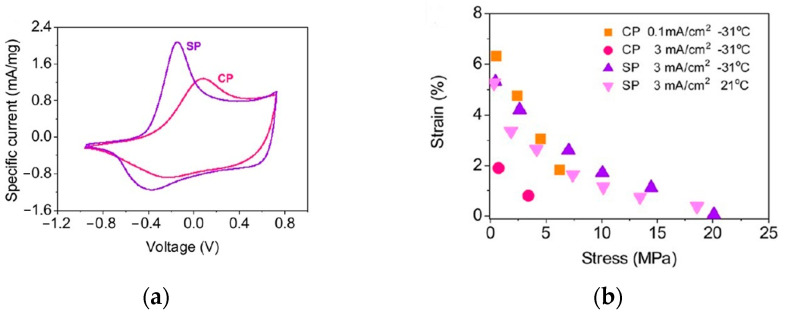
(**a**) Cyclic voltammograms for CP (continuously polymerized) and washed SP (sequential polymerization) PPy films prepared at 3 mA∙cm^−2^ and −31 °C; (**b**) actuation strain at different applied stresses for films (reprinted with permission from [59]. Copyright: 2024 American Chemical Society).

The low mechanical strength and modulus of the CP films stem from several parameters, such as a high degree of porosity, defects, and rough surface. The pores and internal defects behave as stress concentrators or plasticizers. Spinks et al. obtained a material with high conductivity and superior mechanical properties via modification of the synthetic path, where several washing steps were added [59]. The alternative procedure enabled a decrease in the concentration of oligomeric species in close vicinity to the electrode, hence hindering three-dimensional growth, leading to a more compact layer. The lack of these obstacles led to a highly conducting, smooth film with improved mechanical characteristics (Figure 8), which is highly desirable in artificial muscle operation [59,97]. The actions undertaken to improve the behavior of coatings involve modification of deposition process itself or introducing novel additives that introduce expected modification.

**Figure 9:**
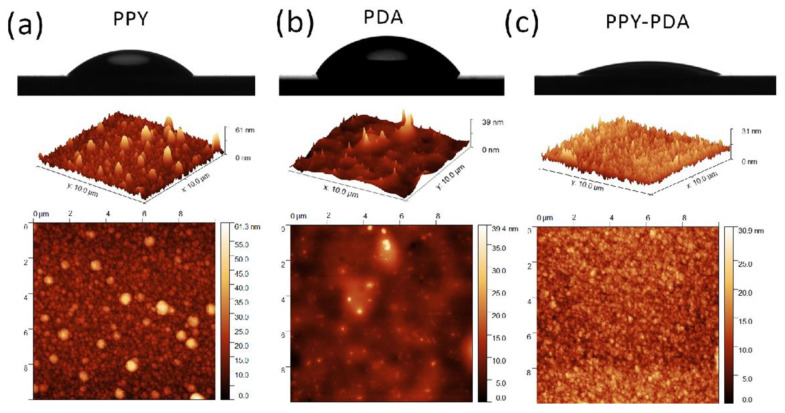
The water contact angle and AFM characterization of (**a**) PPY, (**b**) PDA, and (**c**) PPY-PDA coatings deposited on planar Au/Si electrodes (adapted from [98] under the terms and conditions of the Creative Commons Attribution (CC BY) license).

In the work of Nautiyal [27], adhesion was improved by adding an organic modifier, namely, decanoic acid (DA), that served as an adhesion promoter by forming an anchor in the metal–coating interface [27]. Attempts to improve the adhesive properties and biocompatibility of PPy were also realized via the incorporation of biomolecules like polydopamine (PDA) [98]. Its presence increased the rate of hybrid film deposition, along with adhesion and surface homogeneity, achieving efficient charge-transfer properties. Hybrid PPy-PDA is composed of agglomerates (Figure 9), with the R_q_ value of 3.2 nm being the intermediate value of PPy (5.0 nm) and PDA (2.4 nm). In hybrid film, improved film homogeneity and increasing hydrophilicity were manifested [98]

**Figure 10:**
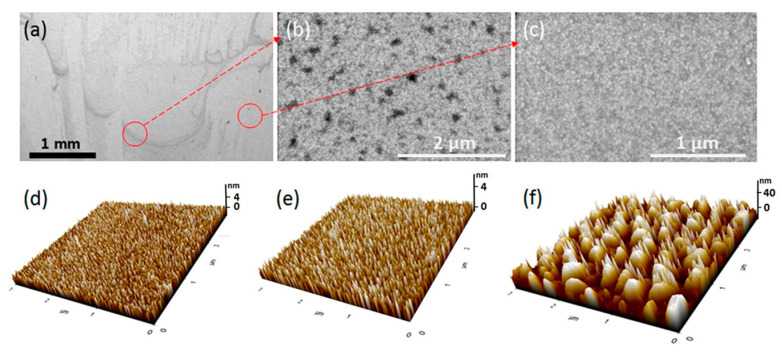
The PEI-O2 Au electrode: (**a**–**c**) scanning microscopy images at (**a**) a low magnification and (**b**,**c**) high magnification; atomic force microscopy images of (**d**) bare Au, (**e**) PEI-O2 Au, and (**f**) thick polymer-coated Au (adapted from [100] under the terms of the Creative Commons CC BY license).

A modified approach to enhance corrosion-protection capabilities via the use of CP coatings was proposed by Mahdavi [99]. In this study, the polypyrrole matrix was enriched with the hydroxylated barium titanate nanoparticles (BT-OH) functionalized with PDA. The role of the PDA was to promote the dispersion of nanoparticles. The deposition of PPy on NiTi alloy in the presence of PDA-functionalized BT was conducted potentiostatically. After introducing PBT nanoparticles, the surface morphology of the polymer evolved from cauliflower-like to nodular-like. Surface roughness parameters, including the arithmetic average of the roughness profile (R_a_), root-mean-square average of the roughness profile (R_q_), and maximum peak height above the mean line (R_p_) for the coated NiTi specimens, were determined with Ra for a PPy-coated specimen equal to 35.5 nm. In contrast, for the composite coatings, it increased from 44.8 to 83.8 nm as the content of the PBT was higher. Electrochemical tests in the Ringer solution demonstrated higher corrosion protection and lower Ni ion release for composite-coated NiTi [99]. A CP-coated gold electrode with enhanced interfacial adhesion strength was proposed by Sung Yeol Kim [100]. The interaction between substrate and polypyrrole was enhanced by the introduction of a polyethyleneimine (PEI) adlayer (Figure 10). The cohesion of the PPy layer was stronger than the interfacial adhesion between the PEI and PPy layers. Mechanically stable layers (also at a negative potential sweep) retained electroactivity, with an R_a_ value of 11.52 nm for thick film deposited potentiostatically.

A roughness-based procedure was proposed to achieve non-delaminating coating on Au electrodes [95]. In the study, the surface of the substrate was deliberately roughened either by electroplating (Au plating) or by etching (with a commercial wet gold etchant). The results of a tape test that quantified the extent of delamination showed that PPy doped with dodecylbenzene sulfonate ion (DBS^−^) delaminated markedly between 1 cycle and 20,000 cycles, while both methods of roughening the surface improved the adhesion and lifetimes to at least 10,000 cycles [95]. An electroplated gold adlayer, an adhesion promoter for PPy coatings, was also reported by Cui [101]. The polymer deposited on treated surfaces remained attached to the electrode 5 times longer than the untreated substrates. Adhesively bonded polypyrrole thin films doped with benzene sulfonic acid (BSA) were electrodeposited on aminobenzenediazonium-modified flexible ITO electrodes [16]. The adhesive layer was grafted to the substrate via electroreduction of the in situ-generated diazonium compound. Subsequently deposited polypyrrole thin films improved adhesion to aminophenyl-modified ITO [16]. The SEM microphotographs of bare ITO, ITO-AP, and ITO-AP-PPy electrodes showed evolution from a rough surface with scattered aggregates for a bare ITO surface, a nanostructured network for an ITO-AP functionalized surface, to cauliflower agglomerates of an electrodeposited PPy-BSA layer, which formed continuous and compact film. The device was tested as a sensor to detect selected metal ions in an aqueous medium. A similar successful strategy was utilized for polypyrrole deposition on a Nitinol substrate [102] in the photopolymerization process. Electrodeposited PPy/DBS layers improved porous sintered stainless steel’s protection against corrosion in work by Garcia-Cabezon [103]. More homogeneous and stable coatings were deposited for porous stainless steel substrates than for wrought ones, with corrosion potential values shifted to nobler values and a more stable anodic polarization branch. Duplex polypyrrole films electrochemically synthesized on iron were tested as protective coatings against corrosion [104]. The inner film included doped tetraoxalate anions, while the outer film featured dodecyl sulfate anions. The microroughness of PPy/DS provoked favorable action in terms of the improved adhesion of a top coating on such a film. The outer film had the major effect of preventing the ingress of chloride anions. In the test, the permselectivity of the film was changed from anionic to cationic, preventing the ingress of corrosive chloride anions.

**Figure 11:**
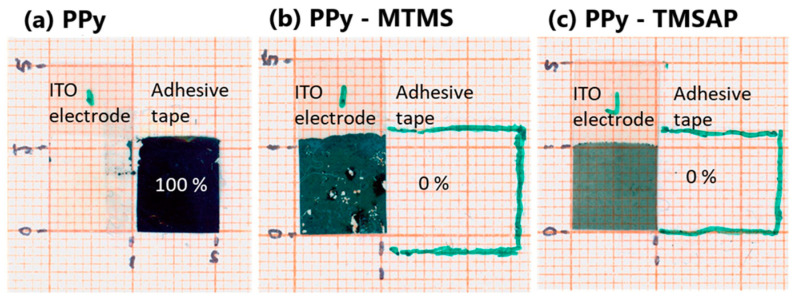
Scotch-tape adhesion test of polypyrrole films: (**a**) PPy film control sample and (**b**) PPy-MTMS and (**c**) PPy-TMSPA films. In samples (**b**,**c**), the boundary of the transparent adhesion test is outlined with green to make observation easier (adapted from [106], distributed under the terms and conditions of the Creative Commons Attribution (CC BY) license).

Polypyrrole films deposited on stainless steel with anodic electropolymerization in the presence of sulfosalicylic acid (SSA) adhered well to the substrate. The adsorption of an anionic dopant blocked the anodic oxidation of stainless steel at the polymerization stage, providing conditions for highly adherent film formation [105]. Moreover, in the same work, sodium salt from poly[2,5-bis(3-sulfonatopropoxy)-1,4-ethynylphenylene-alt-1,4-ethynylphenylene] dispersed single-walled carbon nanotubes (SWNTs), enhancing homogenous composite PPy–SWNT formation, which was characterized by increased porosity and improved capacitive behavior [105]. The effect of the alkoxysilanes (namely, methyltrimethoxysilane (MTMS) and N-[3-(trimethoxysilyl)propyl]aniline (TMSPA)) on the topography of the pPy film was studied by Castro-Beltran [106]. Pure pPy film samples showed high roughness and globular aggregates on the surface. The addition of silanes at the synthesis stage resulted in films with smoother surfaces and the growth of small globular features, improving adhesion (Figure 11). The authors concluded that in the presence of additives, Py electropolymerization is hindered due to reduced monomer oxidation, resulting in less polymer deposition than the control sample. A lower deposition rate led to a smoother surface, which suggests more ordered film growth due to slower polymerization.

On the other hand, delamination of the polymer coating was proposed to obtain free-standing foil. Free-standing PPy films doped with dodecylbenzene sulfonate (DBS) obtained via electrochemical delamination were thinner than films obtained using mechanical peeling [107]. The substrate side of the PPy/DBS films mirrored the substrate surface roughness (either 1 μm polished 304SS or gold foil). The solution-side surface of the films showed a nodular morphology, with nodules forming clusters with diameters of 1.6–13.7 μm. The larger cluster diameters observed for 304SS compared to gold (for the same charge densities) are related to faster film growth on 304SS. The filling efficiency, which quantifies cation transport in the deposited films, decreased with increased electropolymerization charge density. This was correlated with the rise in the thickness of the film, which hampers the ion-transport process, leaving part of the film underutilized [107,108].

### 5.2. The Role of Roughness in the Cell-Adhesion Process

**Figure 12:**
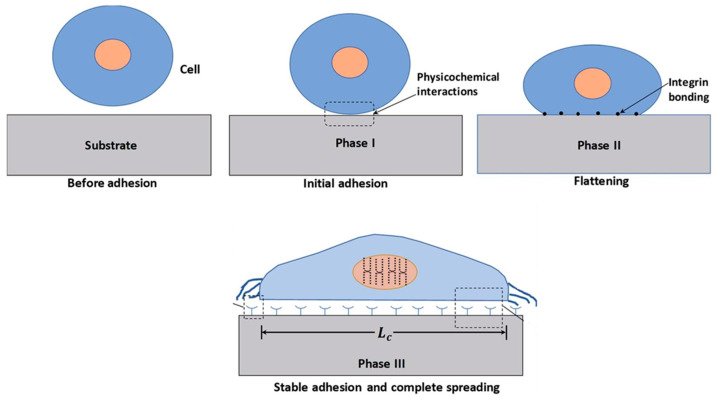
A schematic illustration of cell–substrate interaction from a non-adherent state to the stage of cytoskeleton organization with stable adhesion (adapted from [109] under a Creative Commons Attribution—Noncommercial 3.0 Unported Licence).

For biomedical applications, it is urgent to determine the cell-adhesion behavior, including adhesion to the coating. This process involves three stages, as illustrated in Figure 12. Cell–substrate interaction evolves from a non-adherent state to the stage of cytoskeleton organization in stable adhesion provoked by multiple adsorption events [109]. The process of cell adhesion is governed by the cell’s interaction with external surfaces, in particular with chemical (the surface energy of the substrate [110], topological, and mechanical factors. It was typically found that cells prefer to grow on higher energy surfaces either by the formation of chemical bonds with the polar groups on polymer surfaces or by short-range interactions created with non-polar groups [111]. Surface topographical factors, including the roughness and porosity of polymer surfaces, also play an urgent role in cell–substrate interactions [112].

There is an increasing demand for materials that can interfere with biological materials, providing applications like biosensors, medical implants, neural interfaces, and tissue engineering scaffolds [77]. Depending on the desired application, there are efforts to design either materials and surface architectures that inhibit the growth of a wide range of bacterial species simultaneously or engineer biocompatible surfaces.

An improvement in cell affinity and bioactivity of PPy was reported by Ge [113] by producing a nanocomposite with bioactive PDA on monocrystal silicon wafers coated with titanium and gold (Au/Ti/Si wafer) as a substrate. Using two-step synthesis, a functional conductive coating was deposited, with PPy nanowires used as the morphologic support layer and an outer layer of bioactive PDA. Compared to pristine PPy NWs, the surface roughness of PPy/PDA nanowires increased, while the PDA adhered to the surface of PPy nanowires, leading to a PPy/PDA core–shell structure. The hydrophilic character of the PPy/PDA composite (water contact angle of 16°) was prescribed to the rough, porous nanostructure and a large number of exposed hydrophilic groups on the surface of PDA [105]. Such characteristics improved the adhesion, proliferation, and osteogenic differentiation of MC3T3-E1 cells cultured on this surface. The surface characteristics of PPy concerning biomedical applicability were studied by Schmidt [114]. The electrochemically synthesized films were deposited on two substrates (gold and ITO-coated glass) in the presence of three dopants (chloride (Cl), tosylate (ToS), and polystyrene sulfonate (PSS)). There were significant differences in roughness parameters depending on the dopant used and the thickness of the film. The PSS-doped film was the smoothest one, with the material deposited evenly across the substrate, presumably because of the size of the large polyanion dopant adsorbing at the electrode and providing an enhanced surface for subsequent PPy adsorption. Cell-adhesion studies indicated the dependence of cell outcome on film thickness and dopant choice, with Cl- dopant being promising [114]. The study proved that different dopants yield vastly different material properties, like high conductivity (ToS-doped PPy), maintaining composition over a long time with a smooth surface (PSS-doped PPy), and high surface roughness (Cl-doped PPy).

**Figure 13:**
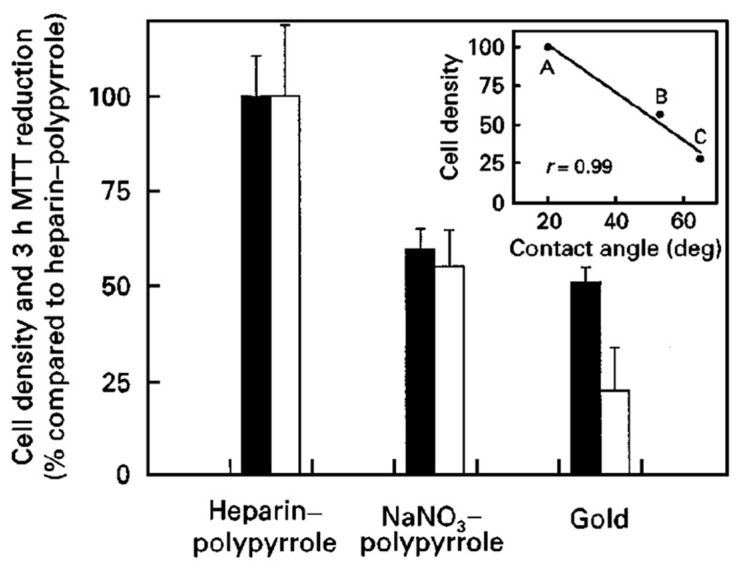
The number of HUVEC cells present (white bars) or the amount of MTT reduced in 3 h (black bars) was assessed after 90 min for doped PPy and a gold substrate. Inset: the correlation of the cell density versus the hydrophobicity of the samples for A (heparin-polypyrrole), B (NaNO_3_-polypyrrole), and C (gold) (adapted from [116] under a license agreement).

A method of achieving more advantageous properties, including antifouling behavior, stimuli-responsive character, and the ability to modulate cell growth and differentiation, is functionalization. CPs modified to promote cell adhesion were proposed by Lee [115] via the synthesis of amine-functionalized polypyrrole (APPy), which presents cell adhesion-supporting positive charges under physiological conditions. APPy deposited on a gold-coated glass slide exhibited superior attachment of human dermal fibroblasts and Schwann cells compared to PPy homopolymer under serum and serum-free conditions. In this case, the roughness of regular PPy and APPy films was similar without substantial differences, which proved the lack of the topographical effects of APPy on cell adhesion. The amine-functionalized films were proposed as a substitute for conventional polymeric cations (e.g., poly-L-lysine (PLL)) used to promote cell adhesion. A composite composed of PPy with biologically active polysaccharides, namely, heparin grown galvanostatically on gold-coated mylar films, was studied by Hodgson [116]. The composite was intended for use as a substrate for human umbilical vein endothelial cell (HUVEC) growth. It was suitable for supporting cell attachment and growth, with a smooth surface of R_q_ equal to 11.8 nm (for oxidized film) and 10.5 nm (for reduced film). Doubling times for HUVEC on heparin-polypyrrole were longer than for gelatin-coated polystyrene tissue culture (44 vs. 36 h). The surface hydrophobicity and roughness are known to influence endothelial cell attachment to biomaterials (Figure 13). In this work, an inverse linear correlation of the number of cells attached to the materials compared to their hydrophobicity (expressed as the contact angle) was reported.

**Figure 14:**
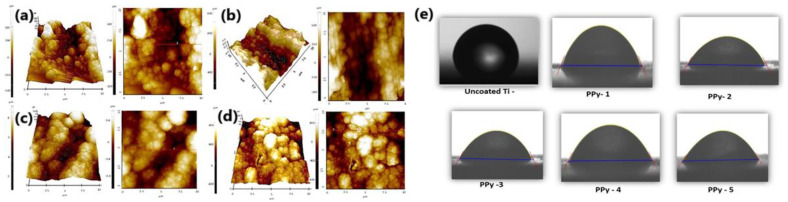
AFM images of (**a**) PPy-1, (**b**) PPy-3, (**c**) PPy-4, and (**d**) PPy-5 with (**e**) corresponding water contact angles. The coatings deposited with various concentrations of Py, namely, 0.1 M, 0.2 M, 0.3 M, 0.4 M, and 0.5 M, were denoted as PPy-1, PPy-2, PPy-3, PPy-4, and PPy-5 (adapted from [119] under a license agreement).

The application of a small potential bias (+200 mV) to a PPy-HA film had a large effect on the early attachment and spreading of Human Adipose Stem Cells (HASCs) [117]. The polymers were electropolymerized on ITO substrates or Au-Mylar films using the potentiostatic method. Before seeding cells, the PPy-HA samples were gamma-sterilized (a 20 kGy radiation dose). R_q_ roughness values of the uncharged and charged polymer were 8.9 and 9.4 nm, with a minor effect of the electrochemical charging on the surface roughness. The majority of HASCs were uniformly adhered on charged sample surfaces, but on noncharged surfaces, cells were unevenly spread, and many of them did not adhere well. A preincubation period in the cell culture medium containing plasma proteins markedly influenced the efficiency of cell attachment [117]. A surface-modification procedure using organic adlayers was described by Krukiewicz [118], where electrografted diazonium salts were functionalized with biologically active molecules. The proneness of the sample to cell attachment was evaluated by culturing human neuroblastoma SH-SY5Y cells. Cell adhesion was favored on the surface of diazonium-modified and poly-L-lysine-coated Pt electrodes, proving the vulnerability of the modification route. The sample roughness (S_a_) of modified electrodes increased, especially after PLL coating, in comparison to pristine Pt (1.5–2.0 µm vs. 0.019 µm (uncoated)/0.221 µm (PLL-coated)). Titanium (Ti) coated with bioactive polypyrrole was studied in terms of its anti-corrosion protection and biocompatibility [119]. The deposition process performed in the CV mode led to typical cauliflower-like structures. The irregular PPy deposits with the variable sizes of the grains led to the R_a_ parameter value ranging from 45 to 57 nm (changing along with the monomer concentration) compared to 32 nm for the uncoated substrate (Figure 14). This parameter influenced the bioactivity and bonding ability with more cells attached and improved the osseointegration behavior for coated implants. This was also a reflection of the more hydrophilic character of the coated Ti surface [119].

**Figure 15:**
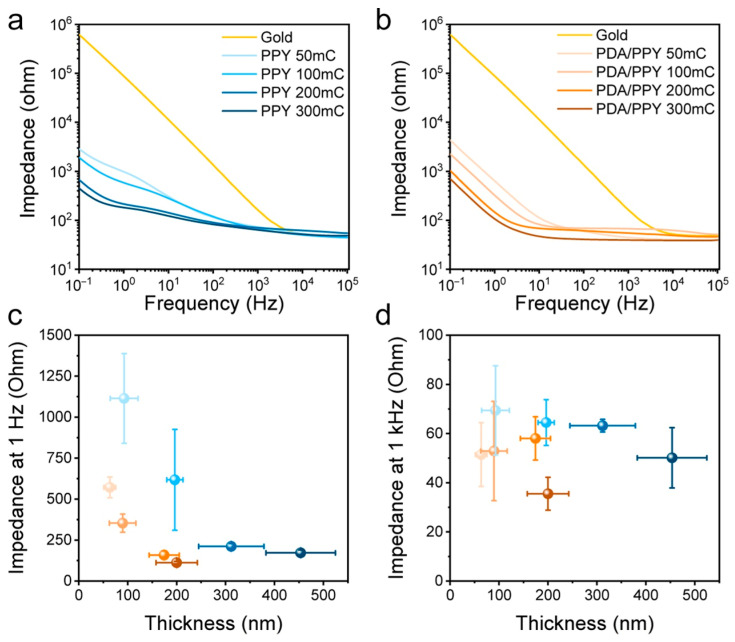
The electrochemical properties of PPy and PDA/PPy: the bode plots of (**a**) PPy- and (**b**) PDA/PPy-modified gold electrodes prepared with different deposition charges. Thickness–impedance plots of PPy- and PDA/PPy-modified electrodes at (**c**) 1 Hz and (**d**) 1 kHz, respectively. Reprinted with permission from the authors of [120]. Copyright: 2024 American Chemical Society.

The electrochemical copolymerization of polydopamine and PPy performed for electrode modification was studied by Lee et al. [120]. In the ED step, a gold substrate served as a working electrode, while a galvanostatic method was used with several charge densities applied to prepare different coating thicknesses. The topographies of coatings were affected by deposition charges, as thin coatings were smooth, and increased deposition charges led to roughening being more intense for a homopolymer coating (the R_a_ of PPy was 40.27, while for PDA/PPy, it was 8.55 nm). An increase in the thickness of the polymer coating led to a decrease in the impedance values with the impedance at 1 Hz and 1 kHz. These frequencies are commonly utilized to determine the usability of materials as they cover the scope of the biologically important frequency range for electrical stimulation of muscle/nerve tissue [121]. The impedances of PDA/PPy electrodes were markedly lower compared to the PPy ones, with increased capacitive properties of the electrodes (Figure 15). For both C2C12 myoblasts and PC12 neuronal cells, the growth and differentiation of PDA/PPy were enhanced, while the electrical stimulation of neural cells on a copolymer promoted neuritogenesis [120].

**Figure 16:**
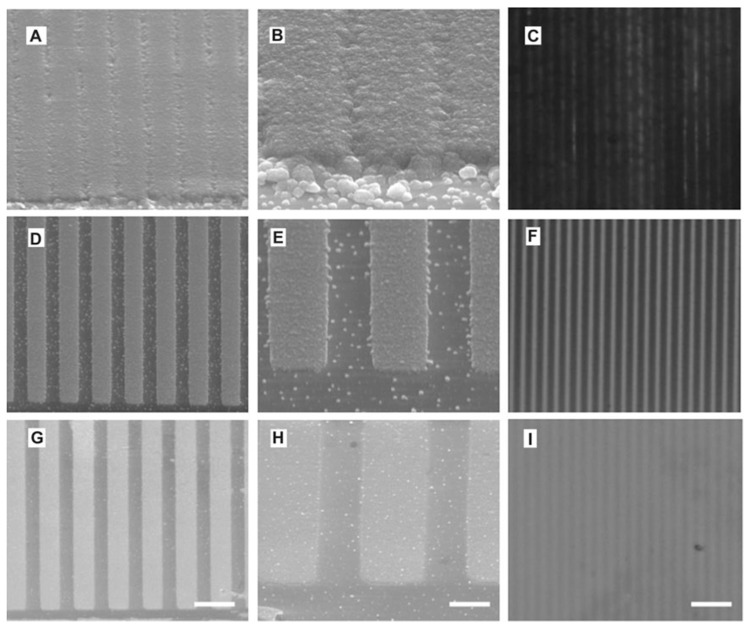
The PPy microchannel morphology in different electropolymerization conditions: (**A**,**B**,**D**,**E**,**G**,**H**): SEM images; (**C**,**F**,**I**): light microscopy images. Microchannels polymerized within 30 s and: (**A**–**C**): 50 mM of pyrrole (thickness ca. 900 nm); (**D**–**F**): 25 mM of pyrrole (thickness ca. 300 nm); (**G**–**I**): 12.5 mM of pyrrole (thickness ca. 50 nm) (adapted from [122] under a license agreement).

To trace the effects of topography on the polarization and axon elongation of neural cells (namely, embryonic hippocampal neurons) cultured on modified substrate, PPy microchannels were fabricated [122]. The system was prepared using a staged procedure, first by patterning an insulating resist on a conductive substrate using electron beam lithography and subsequently by polymerizing on the exposed conductive areas. The topography of PPy microchannels depended on electropolymerization conditions, including monomer and dopant concentrations, the polymerization current, and time (Figure 16). Larger polymerization currents and higher monomer/dopant concentrations led to fast polymerization; hence, obtained structures were deeper, rougher, and provided less defined geometries while decreasing the gap distance between subsequent microchannels [122].

### 5.3. The Role of Roughness in Protein Adhesion—Antibacterial Coatings

**Figure 17:**
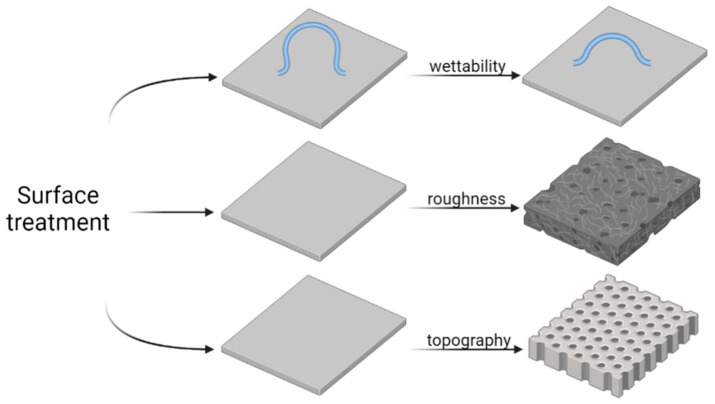
A schematic representation of surface treatment strategies used to modulate bacteria adhesion and biofilm formation by changing surface properties, namely, wettability, roughness, and topography (adapted from [125] under the terms and conditions of the Creative Commons Attribution (CC BY)).

The interactions between biomaterials and bacteria proceed in several stages, with an interaction phase at the beginning and a subsequent molecular and cellular one [123]. It was proposed that the irregular polymeric surfaces enhanced bacterial adhesions, contrary to the ultra-smooth surface [124], and enhanced surface roughness provided more accessible sites for bacterial colonization. Moreover, some deliberate mechanical processes (e.g., grinding, polishing, sand-blasting, sintering, heat treatment, and etching) [37,125] and ion-implantation technology [126,127] that generated surfaces with anti-bacterial properties were proposed (Figure 17).

The antibacterial behavior of PPy depends on a diversity of structural parameters, such as the surface area, aggregation level, and incorporated additives (e.g., metal nanoparticles) [123,128]. These properties are related to polymerization solution compositions and conditions; hence, the final material characteristic compromises between them [126]. The antibacterial-inhibition mechanism relies on electrostatic interaction between the positive charges on the polymeric chains and the negative charges on the membrane cell of the bacteria. Moreover, an attack on the cell wall of the bacteria via a charged N atom and dopant ions of the polymers is possible, leading to bacterial cell death [123].

The effects of bacterial adhesion on PPy films synthesized on gold-coated silicon chips were tested by Jager [129]. Five types of dopants, namely, chloride (Cl^−^), perchlorate (ClO_4_^−^), p-toluene-sulfonate (ToS), dodecylbenzene sulfonate (DBS), and polysodium styrene sulfonate (PSS), were used for synthesis at two different constant potentials (0.500 and 0.850 V) with and without Fe^3+^ ions. The roughness could be tuned by utilizing the proper counter ion, synthesis potential, consumed charge, and presence of Fe^3+^. The surface properties of the films affected bacterial adhesion. Films with higher roughness attracted more bacteria than smoother ones (PPy/DBS vs. PPy/PSS). This was correlated with increased hydrophobicity of the surface. In general, the surface properties of polymers were tuned both statically via the choice of the dopant and dynamically by altering the redox state of the PPy. In the sulfosalicylic acid (SSA)-doped polypyrrole, drug molecules were adsorbed on the surface of monomer nano-droplets, forming a stable micellar system [130]. Their presence introduced hydrogen-bonding interaction between monomer drops and provoked tubular organization of polymers on the rough layer of medical titanium. The presence of SSA enhanced the surface potential of the polymer, enforcing its antibacterial activity. For a PPy/salicylate (SAL)-doped system deposited on carbon steel, salicylate ions inhibited bacterial colonization due to their antibacterial activity. This process was favored by the electrostatic interaction between polypyrrole and the bacterial cell wall, while the presence of the silver layer reinforced the effect [124]. Interestingly, coating adherence decreases with the increase in the applied current density (tested in the standard Sellotape test), proving a difference in the interface formation of a polymer/electrode.

**Figure 18:**
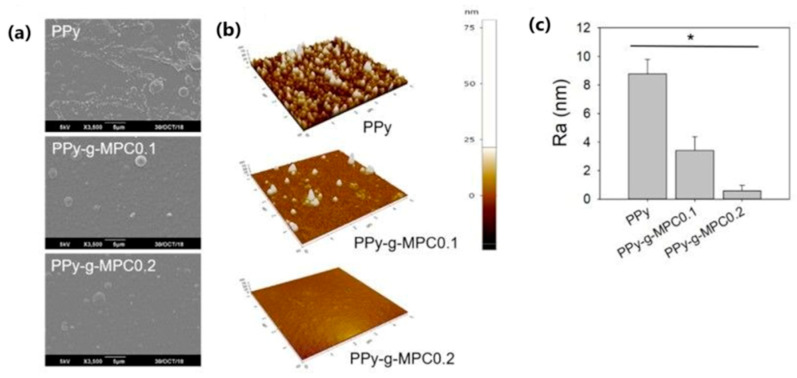
The surface topographies of PPy and PPy-g-MPC samples: (**a**) SEM images and (**b**) AFM images of the samples; (**c**) R_a_ values obtained via AFM analysis, * denotes a significant difference of *p* < 0.05 (adapted from [132] under a license agreement).

On the route to decrease the corrosion rate in physiological media and reduce the risk of implant-associated infections, a magnesium alloy (namely, AZ31 Mg) was covered by a polypyrrole/gallic acid (PPy/GA) coating [131]. Electropolymerization in the potentiostatic mode (0.95 V vs. SCE for 5 min) produced PPy and PPy/GA layers of approx. 26 μm and 23 μm thicknesses. A honeycomb lattice structure rich with cracks was detected on the surface of Mg-PPy film, while GA doping changed the topography to pellet-like. The adhesion test pointed out that the presence of GA improved the adhesion strength of the coating to the substrate [131]. The impact of the roughness of PPy on Ti6Al7Nb alloy on cell activity and cytokine secretion was demonstrated by Mindroiu [84]. The studies of RAW264.7 macrophage cultures on these samples showed the dependence of cell adhesion, proliferation, cellular morphology, and cytokine secretion on the type of surfactant used. Macrophage proliferation was promoted on PPy, PPy/Triton, and PPy/DM surfaces but was handicapped on PPy/PSS surfaces [84]. A novel attitude for the production of non-biofouling bioelectrodes was presented by Lim [132]. PPy deposited galvanostatically on gold electrodes was subsequently grafted with methacryloyloxyethyl phosphorylcholine (MPC) in an in situ polymerization process initiated with gamma radiation. Grafting strategies did not cause impairment in the electrical/electrochemical properties of the original PPy electrodes, imparting anti-biofouling properties. The surface of PPy electrodes was rough (8.8 nm), with multiple spherical nodules and grains (Figure 18). The presence of MPC led to smoother surface enhancement for more heavily grafted samples (3.4 nm and 0.6 nm). The decrease was attributed to the uniform introduction of MPC moieties onto the PPy surfaces, leading to a uniform charge distribution followed by charge neutrality due to two opposite charges, which was desired to support the nonfouling behavior of grafted zwitterionic polymers.

**Figure 19:**
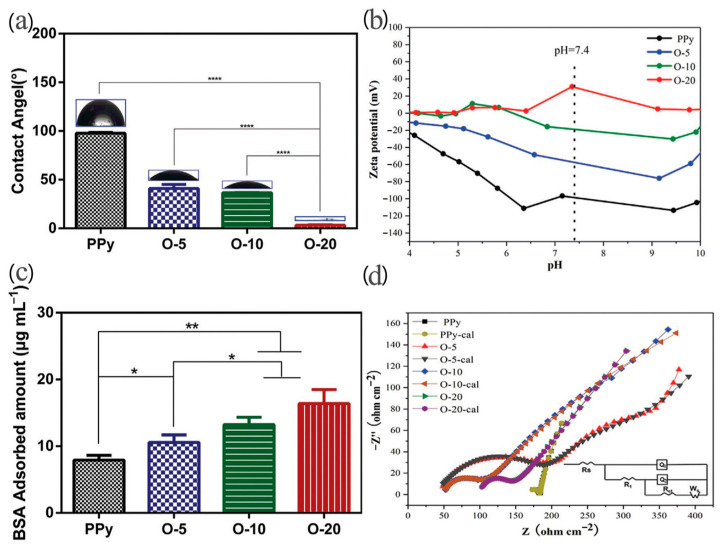
(**a**) The contact angle of PPy, O-5, O-10, and O-20; (**b**) the zeta potential versus pH acquired from PPy, O-5, O-10, and O-20; (**c**) the adsorbed BSA amount of PPy, O-5, O-10, and O-20; (**d**) EIS Nyquist plots of PPy, O-5, O-10, and O-20 and the simulation calculation plots. * *p* < 0.05, ** *p* < 0.01, **** *p* < 0.0001 (adapted from [126] under a license agreement).

The surface morphology, wettability, and surface charge of polymer substrate can be tailored using ion-implantation technology [126,127], with severe effects on the coating’s antibacterial properties. PPy films deposited electrochemically on titanium surfaces with subsequent oxygen plasma immersion ion implantation were the subject of the work performed by Li [126]. The surface roughness increased significantly after an extended implantation time with R_q_ values of PPy, O-5, O-10, and O-20 equal to 68, 172, 210, and 307 nm, respectively, and R_a_ values equal to 48.1, 138, 175, and 231 nm, respectively. The protein-adsorption ability was tested using bovine serum albumin (BSA) as a probe. The highest BSA adsorption amount was found for the mostly implanted sample, while the lowest was found for the unmodified PPy sample (Figure 19). The authors correlated the findings with the hydrophilicity of the surface (the greatest for mostly implanted samples) and enhanced surface zeta potential [126].

### 5.4. Implication of Roughness from Drug-Release System Perspective

**Figure 20:**
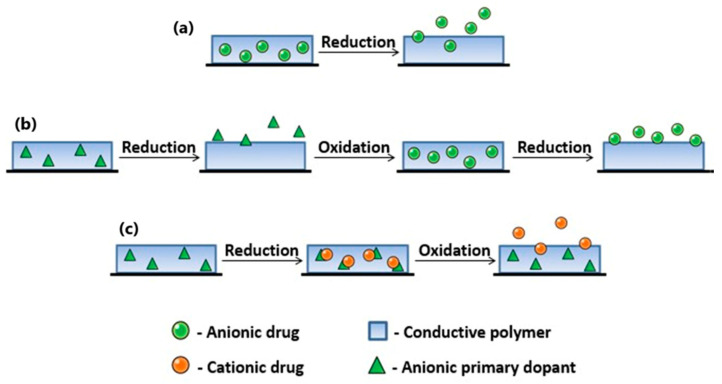
Drug-loading and release mechanisms in CPs: (**a**) one-step loading (for anionic drugs); (**b**) three-step loading (for anionic drugs); and (**c**) loading of a cationic drug (adapted from [133] under a license agreement).

Drug-releasing systems are willingly constructed to release a dose of active substance in a programmed manner. Their utilization helps obtain the therapeutic concentration level for the drug but avoids reaching a toxic level. CP-related drug-delivery systems utilize the ability of the polymer matrix to inhale or exhale the molecules/ions under electrical stimulation, which provokes a transition between an oxidized and a reduced state [133]. The drug-loading procedure depends on the type of the drug molecules, with a one-step immobilization procedure (a) for small anionic compounds or a three-step method (b) where synthesis and drug-loading stages are separated and a modified three-step method for cationic drugs (Figure 20).

**Figure 21:**
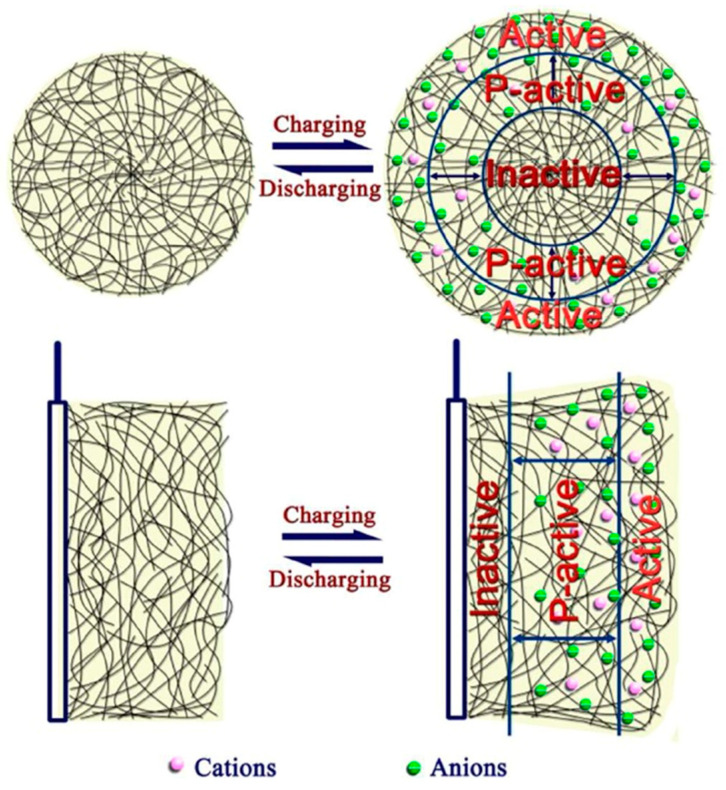
A scheme of a PPy film during the charging/discharging process with inactive, P-active, and active zones (adapted from [108] under a license agreement).

Small anionic drug molecules might be incorporated as dopants in a one-step immobilization process along with monomer oxidation [134]. Nevertheless, the character of the molecule strongly interferes with the final properties of the matrix, like decreasing conductivity or increasing brittleness/roughness, resulting in a low loading capacity. As a solution, a modified procedure for enhanced loading capacity was proposed, composed of a three-step methodology: a pristine polymer synthesized with a virgin anionic dopant, followed by a reduction step aimed at anion expulsion with the use of external potential and an oxidation step aimed at chosen anion incorporation with matrix oxidation [135]. During the electrochemical doping/dedoping cycles, when the ions from the supportive electrolyte diffuse to the polymer matrix, the arrangement of the chains influences the ions’ mobility [108]. More crystalline regions of the matrix improve the electronic and ionic conductivity as a more regular molecular chain provides more stable and smooth channels for the insertion/extraction of ions. In deeper regions, however, the ions from the electrolyte cannot reach the inner part of the matrix owing to a long diffusion path or more compact structure. Hence, the polymer matrix might be treated as if composed of several zones: the inner inactive zone, the intermediate partially active zone, and the outer active zone accessible to the moving ions (Figure 21) [108]. In the field of drug-doped polypyrrole, there is a massive impact of the roughness on the performance of the system. AFM-based investigation of the surface roughness of PPy films indicated that, usually, increasing the deposition charge density led to an increase in roughness and, hence, the dosing of entrapped molecules [135,136].

**Figure 22:**
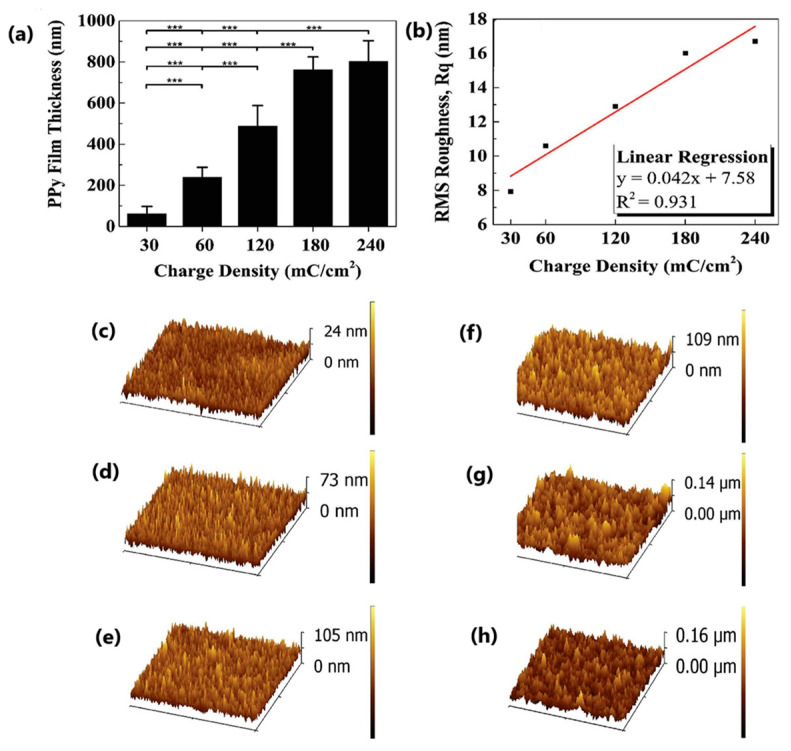
The thickness and roughness of PPy MCs: (**a**) the PPy film thickness as a function of the applied charge density, and (**b**) R_q_ as a function of the applied charge density, The symbol *** indicates a significant difference of p < 0.001. (**c**–**h**) Atomic force micrographs with the R_q_ of the bare gold surface and PPy films produced at 30, 60, 120, and 180 mC·cm^−2^, respectively (adapted from [140] under a license agreement).

The release of substances from CP matrices is dictated by electrostatic forces accompanied by expansion and contraction movements induced by the electro-chemo-mechanical response [137]. Cui investigated the impact of the topography of substrate electrodes on electrically controlled drug release from PPy [138]. Fluorescein acted as a model drug serving the role of the doping ion, while gold electrodes were covered with platinum to elevate the surface roughness of the substrate. The characteristic parameters, like the release per charge accumulation used during polymerization or release per charge injected during electrical stimulation, confirmed increased drug release from a material characterized by higher roughness [138]. Oxacillin-doped polypyrrole film deposited on the surface of platinum and titanium was studied by Moloney [139]. The roughness in the work of smooth polymer films was obtained, ascribed to the equilibrium between the oxacillin anion, Ox−, and the corresponding neutral acid form, HOx, at the polymer solution interface and the lower solubility of HOx. Still, the cross-sections of the polymer revealed a more porous internal structure. The cross-section-based analysis led to the film thickness of 1.50 μm for PPyOx (deposited to 0.25 C). Improved adhesion for the reduced PPyOx was reported for a system enriched with an additional chitosan layer [139]. PLGA microsphere templates were used to produce PPy microcups, with the surface roughness (R_q_) (determined using atomic force microscopy) increasing gradually with the deposition charge density (Figure 22) [140]. R_q_ of the reference bare gold electrode was 2.38 nm with 7.93–16.7 nm for deposition charge densities of 30–240 mC·cm^−2^, respectively. The larger surface area and higher roughness of the PPy deposited at a higher charge density led to a profound increase in the initial burst release of anti-inflammatory prodrug (dexamethasone—Dex), which increased from 66% (at 30 mC·cm^−2^) to 78% (at 180 mC·cm^−2^) [140].

**Figure 23:**
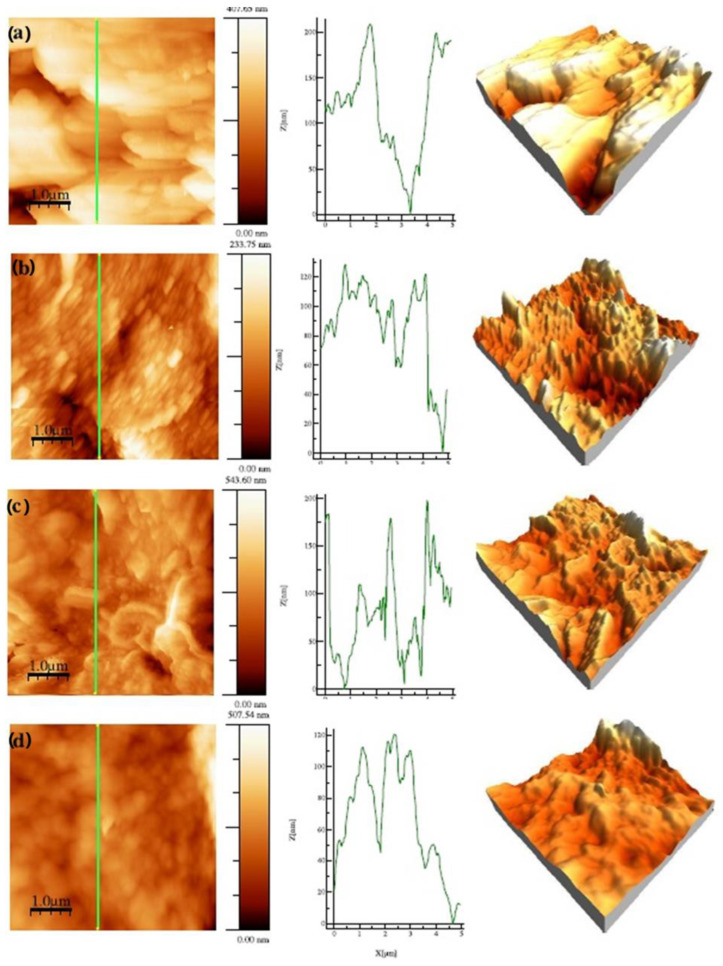
AFM micrographs demonstrated the surface topography of PPy films with n = 5: (**a**) PPyCl–Ti, (**b**) PPy[P/S]–Ti, (**c**) PPy[Dex]–Ti, and (**d**) PLGA–PPy[Dex]–Ti (adapted from [141] under a license agreement).

For nanostructured polypyrrole coated on anodized nanotubular titanium antibiotics (penicillin/streptomycin, P/S) or anti-inflammatory dexamethasone, Dex was proposed as a dopant [141]. PPy films possessed nanometer-scale roughness (Figure 23). SEM and HRTEM analysis revealed that the PPy electrodeposited around incorporated MWNTs with PPy-Ti exhibited a lower surface area (R_q_ = 35.87 nm) compared to PPy/P/S-Ti and PPy/Dex-Ti (R_q_ = 57.58 and 100.04 nm, respectively). The surface of PLGA-PPy/Dex-Ti reached the highest R_q_ among all the samples (198.67 nm). The cumulative release of P/S and Dex was reported as 80% of the drug, while there was no significant drug release without an applied voltage. Moreover, in cell-adhesion studies, fibroblasts adhered more on PPy containing -CO- groups, namely, PPy/Dex-Ti and PLGA-PPy/Dex-Ti, than on PPy containing -NH- groups, namely, PPy/P/S-Ti and PPy–Ti. Studies confirmed that functional groups like amine (NH_2_) and carboxylic (COOH) can modulate fibroblast adhesion.

**Figure 24:**
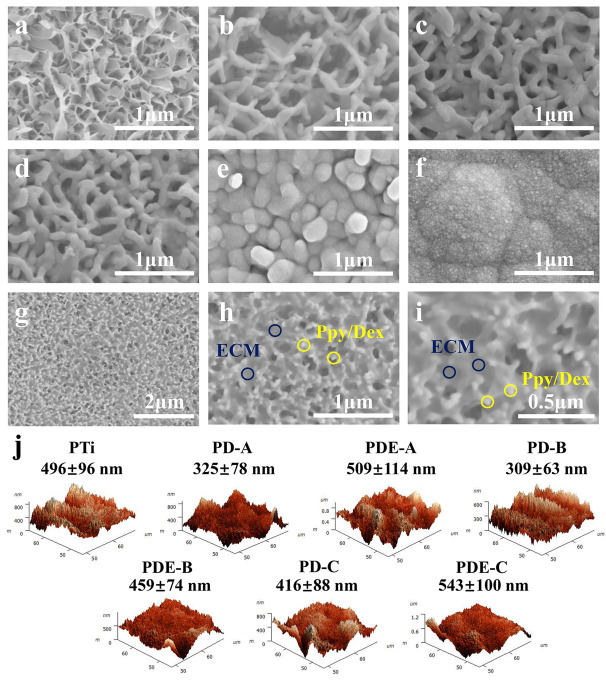
SEM images of (**a**) PTi and PPy/Dex coatings with deposition parameters of (**b**) 0.5 mA/120 s, (**c**) 0.5 mA/300 s, (**d**) 1.0 mA/120 s, (**e**) 0.5 mA/600 s, and (**f**) 1.0 mA/300 s and (**g**–**i**) PPy/Dex/ECM coatings with deposition parameters of 0.5 mA/120 s. (**j**) AFM topography (adapted from [143] under a license agreement).

A hybrid conducting system constructed with PPy doped with dexamethasone phosphate (DexP) was proposed by Moulton and Wallace [142]. Post-surface modification with thiolated poly(ethylene glycol) was applied to enhance polymer biostability. In terms of film topography, the surface roughness of PPy/DexP films increased along with current density growth. The counterion type has a significant influence on polymer topography, as surface roughness significantly decreases with the increase in the DexP concentration. It was correlated with lower film viscoelasticity after increasing the dopant concentration, implying that a higher amount of dopant in the solution affects denser and smoother films [142]. In a study by Chen [143], the porous titanium matrix was coated with polypyrrole/dexamethasone composite coatings in a galvanostatic process. The biocompatibility of the composite coatings was confirmed to be crucial for prospective bone tissue engineering applications. Dex release occurred upon changing the redox state of PPy with negative pulses of the electrical stimulation. The topography of the coatings was influenced slightly by the addition of PPy/Dex or ECM (the extracellular matrix components) (Figure 24). The deposition of polymer in the presence of Dex reduced the roughness resulting from the connection of the pores in the Ti substrate with a porous surface after platinum injection. The presence of ECM increased the roughness up to a value of 300–500 nm, with a minor effect on the electrochemical and biological response.

The impact of large molecules like polysaccharides, e.g., heparin, on PPy synthesized electrochemically was studied [144]. CV was applied as a deposition method on a Au/TiO_2_-coated quartz crystal electrode (Hep, MW = 17,000–19,000 Da). The analysis of AFM images revealed the influence of the dopant concentration on the coating surface roughness. The films were characterized by a dendritic porous morphology, with a roughness of 118 and 58.2 nm for 0.2 and 0.4 Hep content. The more porous structure provides a greater surface area and better access for the molecule-adsorption process. This was proved in a test where Hep present in polymer films was bound to thrombin, with an increased quantity found for coatings with higher surface roughness [144]. The release of an anti-inflammatory drug, namely, salicylate, from PPy-coated iron was reported by Cysewska [145]. It was traced during the degradation of the material previously deposited in a one-step potentiostatic process. The drug-release experiment delivered a quantitative description of salicylate release from PPy-coated iron. The AFM images presented a rough surface with cauliflower-like structures. The observed changes in roughness were relevant to the concentration of sodium salicylate, with higher values obtained for more concentrated solution (S_a_: 80, 100, and 180 nm for 0.1, 0.2, and 0.3 M of NaSal). However, in this case, release profiles recorded during the spontaneous evolution of the drug indicated that the morphology and electrical properties of PPy/Fe exhibited a negligible influence on the release process.

Therapeutic codrug uploading into CP matrices was the subject of work performed by Alshammary [146]. PPy was deposited on an active AZ31 Mg alloy in the presence of ibuprofen and salicylate-containing solutions. A maximum ibuprofen concentration of 440 μg∙cm^−2^ was obtained in PPy films in the presence of sodium salicylate. The presence of IBU influenced the nucleation and growth of PPy films, leading to the formation of cylindrical, needle-like structures. The average film thickness (20 cycles) was ca. 20 μm for both types of films. Furthermore, the incorporation of two anti-inflammatory drugs, dexamethasone phosphate and sodium salicylate, into the electrodeposited PPy film on the iron substrate was investigated [18]. Adding a high-molecular-weight co-dopant influenced the growth mechanism and the drug release profile. The roughest structure was found for the highest concentration of co-dopant, DEX-Na2P (3 mM: S_a_ 95 nm). However, a smaller amount of co-dopant led to a contradictory effect. Such observation proved the impact of the addition of the DEX-P co-dopant on the nucleation and growth of the film on the iron substrate. It also influenced the changes in the electroactivity of films related to the roughness parameters and film porosity. CPs can release drugs in an active (potentially induced) manner, but they can also be applied as passive containers where the loading capacity depends on the porosity of the coating. In work [147], the micro- and nano-gaps between the nanowires of PPy were utilized as reservoirs to store drugs, improving the drug-loading capacity.

### 5.5. Wettability’s Dependence on Roughness

**Figure 25:**
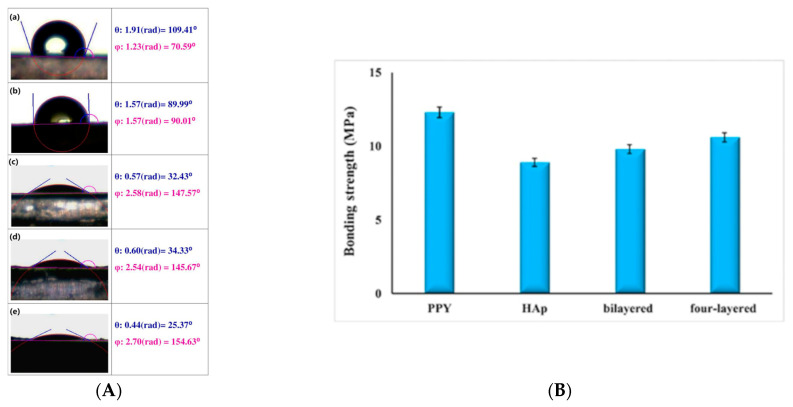
Contact angle measurements for deionized water on (**A**) (**a**): 316 L SS, (**b**): PPY single layer, (**c**): HAp single layer, (**d**): bilayered coating, and (**e**): four-layered coating. (**B**) The adhesion strength of the electrodeposited films (adapted from [154] under a license agreement).

The roughness, geometry, and hydrophobicity of materials strongly influence surface wettability [148,149]. A solid surface with controlled wettability has use in many applications, like self-cleaning surfaces or microfluidic devices. The multiple synthetic paths to produce CPs (chemical oxidative, electrochemical, or vapor-phase polymerization) provide useful tools to control micro- or nanoscale morphology, resulting in surface wettability spanning broadly from superhydrophobicity to superoleophobicity. The properties of CPs allow for tuning of the surface wettability when utilizing several approaches, like the incorporation of doping ions with distinct characters (either hydrophobic or hydrophilic), structuring polymer backbone hydrophilic/hydrophobic balance, as well as the introduction of substituents [148]. Some elegant and practical strategies helpful in wettability modification include the use of hydrophobic doping ions, e.g., 3,5-diisopropyl salicylic acid [150]; the grafting of a substituent on the monomer before polymerization [151]; post-treatment methodology, e.g., silanization [106], using surfactants at the synthesis stage [152]; and deposition on structured surfaces, like anodic aluminum oxide (AAO) templates [153]. For PPy films synthesized electrochemically on two substrates (gold- and indium tin oxide-coated glass) in the presence of three dopants (chloride (Cl), tosylate (ToS), and polystyrene sulfonate (PSS)), PSS was found to provide material with the most hydrophilic surface. This was ascribed to free charges from the long PSS chains exposed on the surface of the PPy [106]. The bioactivity of 316L stainless steel covered with a polypyrrole/hydroxyapatite (Hap)-layered hybrid multilayer system was reported by Mahdavi et al. [154]. The presence of the PPy interlayer resulted in decreased porosity and changed the plate-like surface morphology of Hap. Contact angle measurements for studied samples showed a lower contact angle for the coated substrates than the bare substrate (Figure 25). The surface properties, such as roughness and wettability, profoundly affect the quality of initial interactions between the implant and host tissue for implant application [154].

**Figure 26:**
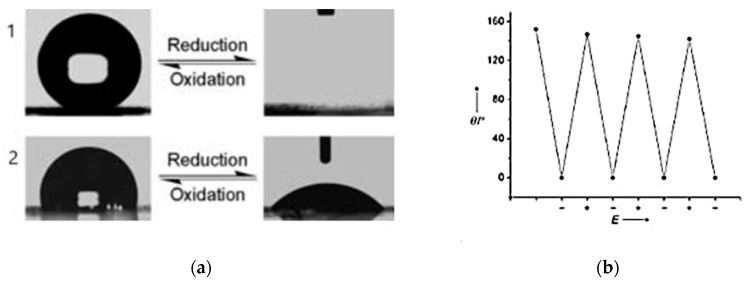
(**a**) The water drop profile on (**1**) a highly porous PPy film, (**2**) a compact PPy film, and (**b**) superhydrophobic-to-superhydrophilic conversion of state for porous PPy films along with alternative negative and positive potential switching; θ = contact angle (adapted from [157] under a license agreement).

To effectively reduce cell adhesion on metallic implants, a pyrrole derivative modified by grafting hyaluronic acid (HA) was studied by Schmidt [155]. The PyHA conjugate was derived by coupling 1-aminopropyl pyrrole to the carboxylic groups of HA. Electropolymerization was performed on Pt, ITO, or polystyrene sulfonate-doped polypyrrole. The surface was characterized as very smooth (for Pt substrate: R_q_ = 2.49 nm), accompanied by hydrophilic properties (θwater ≈ 27–32°), and was unchanged compared to the unmodified electrode impedance characteristics. The p(PyHA) coating has a decreased surface R_q_ parameter value, with roughness attributed to nano-cluster formation. The p(PyHA)-coated electrodes minimized the adhesion and migration of fibroblasts and astrocytes, providing an efficient system against adverse glial responses occurring after implantation [155]. Interaction between an electropolymerized film and negatively charged amphiphilic molecules (AM: sodium dodecylsulfate) was used to adjust the hydrophobicity of the surface of the film [156]. The degree of oxidized CP film surface hydrophobicity was controlled by monitoring the concentration of amphiphilic molecules, while the film surface could be hydrophobically covered by the AMs up to a determined extent. The phenomenon of superhydrophilic/superhydrophobic transition was reversible and repeatable under an applied electrochemical potential. The use of different hydrophobic aromatic molecules (naphthalene, anthracene, pyrene, and a branched aminoalkyl ester) aided in plugging the hydrophilic voids formed on the film surface and increased hydrophobicity. Such behavior is useful for constructing smart clean surfaces exhibiting reversible superhydrophilic to superhydrophobic transitions. Smart surfaces with reversibly switchable wettability were proposed by Yan et al. [157] as superhydrophobic PPy films synthesized on an Au-coated glass by ED in the presence of tetraethylammonium perfluorooctanesulfonate (TEAPFOS, Et_4_N^+^CF_3_(CF_2_)_7_SO_3_^−^). Potential-driven wettability changes were shown for PFOS-doped PPy film in oxidized and neutral (dedoped) states (Figure 26). For highly porous film, distinctly different values of a water contact angle were reported: 152° for the oxidized state and 0° for the neutral state. These values proved superhydrophobic and superhydrophilic natures, respectively. For less porous compact film, the values are less divergent (105° vs. 48°) [157]. Furthermore, Zhu proposed a whelk-like polypyrrole array film synthesized electrochemically in a TEAPFOS solution [158]. The underwater wettability of the material was tuned in repetitive doping/dedoping cycles.

**Figure 27:**
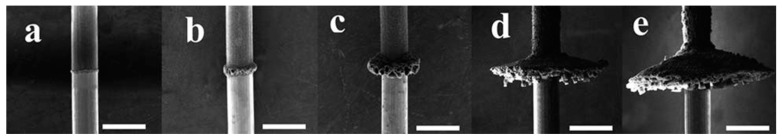
SEM images of free-standing PPy film synthesis. The upper part of Pt is immersed in the water phase, while the lower part is immersed in the organic phase. The polymerization time is as follows: (**a**) 1 min, (**b**) 5 min, (**c**) 10 min, (**d**) 20 min, and (**e**) 30 min. Scale bars correspond to 300 mm (adapted from [160] under a license agreement).

A novel electrode material for symmetric all-solid-state supercapacitors was determined by Zhong [159] by fabricating a large-scale, self-standing polypyrrole/graphene oxide (PPy/GO) nanocomposite. The lamellar structure of GO and mutual strong interaction with ice and pyrrole promoted the polymerization process and improved the compactness of the film. The water contact angle of the composite increased with the increase in GO content, manifesting an increase in the surface roughness of the film. A similar effect was provoked by PPy particles formed on the surface. The symmetric flexible supercapacitors built with PPy/GO exhibited a higher capacitance value (98.4 mF·cm^−2^) at a current density of 1 mA·cm^−2^ compared to pure PPy supercapacitors [159]. A one-step aqueous/organic interfacial electropolymerization produced a Janus free-standing PPy film [160] with a constant current density (Figure 27). Janus objects are characterized with profoundly asymmetric shape and physicochemical properties [161]. In the mentioned work the film roughness geometries differed depending on the phase facing during the synthesis. However, they exhibited superoleophobicity properties, providing a controlled surface wettability product. A phytic acid-incorporated PPy network enhanced the hydrophilicity of the surface prepared as transition metal oxyhydroxide bifunctional electrocatalysts for water splitting [162]. The increased effectiveness of contact between the catalyst and electrolytes manifested as the rise in electrode kinetics for both hydrogen- and oxygen-evolution reactions.

### 5.6. PPy in Microbial Electronics Applications

**Figure 28:**
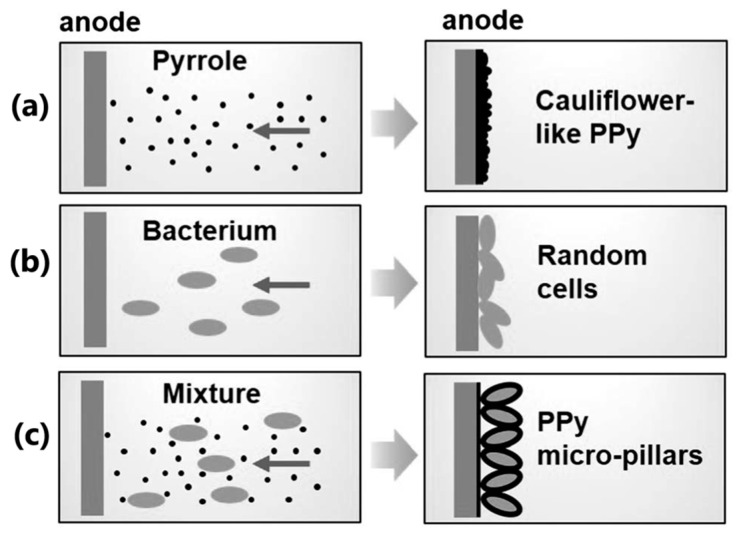
A scheme of Py-MP electropolymerization: (**a**) standard PPy, (**b**) bacterial adhesion to the anode surface, and (**c**) PPy-MP formation (adapted from [165] under a license agreement).

A novel approach using bacteria and a polymer interphase was studied in search of increased energy device efficiency [163]. Bacterial colonies (Shewanella oneidensis MR-1) coated with polypyrrole were used as an anode in microbial fuel cells (MFCs) [164], leading to an increase in direct contact-based extracellular electron transfer as well as the viability of bacterial cells. Furthermore, a method using bacterial extracellular electron transfer was proposed to fabricate oriented polypyrrole micro-pillars (PPy-MP) with nanoscale surface roughness [165]. Microbes were performed as a living template for PPy in situ deposition (Figure 28). PPy of the cauliflower-like structure was synthesized using monomer as the sole reactant. Bacteria led on the anode surface, randomly releasing metabolic electrons utilized for the reaction [166]. The as-prepared film was characterized by distinctive underwater low-adhesion superoleophobicity attributable to hierarchical micro/nano-structures [165]. The tuning of wetting behavior can be observed as cauliflower-like PPy (deposited in the absence of microbes) and exhibited lower surface roughness than PPy-MP (deposited with a living template).

PPy was tested as a novel coating for enhancing microbial charge extraction in microbial electronics [167]. The coated electrodes helped extract photosynthetic electrons from the cyanobacteria, namely, *Synechocystis* sp. PCC6803 (Synechocystis) and Synechococcus Elongatus PCC7942 (Elongatus). A sixfold increase in extracted photocurrent for Synechocystis under unmediated conditions compared to bare graphite electrodes was reported. This enhancement is attributed to the decreased resistance and increased electroactive surface area of the PPy electrode with a globular patterned structure. The increased roughness of the surface may therefore contribute to improved contact and more effective charge extraction from the cells [167].

## 6. Future Development

Based on available data, it is confirmed that the PPy deposition process can proceed rapidly, leading to coating with high roughness and increased porosity. Such coatings may benefit applications like electro-driven drug-delivery systems, porous scaffolds for cell seeding, and electro-driven sorption materials. Conversely, in applications like anticorrosion protection or antibacterial coating, the opposite feature is more beneficial. The final roughness of the coating results from factors that interplay, enhancing the 2D or 3D growth mechanism. In the 2D mechanism, more nucleation sites are formed, allowing for the rapid consumption of available oxidized monomer and reducing the 3D growth of macrochains, providing more compact material. In the 3D mechanism, less effective nucleation occurs, leading to the opposite effect. Awareness of the mechanism allows for the purposeful design of the required structure. The kaleidoscope of ions was tested as a dopant dependent on the application. The chemical nature, as well as their size, were also features that governed the final roughness of the coatings. Organic dopants can especially impart novel effects, e.g., biofunctionalization via hyaluronic acid, while additives like surface-active compounds, e.g., sodium dodecyl sulfate and polyvinyl pyrrolidone, can induce self-assembly interaction, leading to spectacular morphology accompanied by increased adhesion. The presence of various adlayers formed before synthesis (polyethyleneimine, PEI) or at the synthesis stage (polydopamine, PDA) is a validated route of improved adhesion, especially for cell adhesion substrates. The controllable roughness and porosity of the deposited coatings programmed as potentially triggered drug-release reservoirs help increase the drug-loading capacity. Additionally, they enable the tuning of the diffusion of released ions, modifying the shape and time scale of release curves that manifest dosing concentrations. The modern interchangeable surface of various surface wettability can also be constructed by incorporating doping ions with distinct characteristics or structuring the polymer backbone’s hydrophilic/hydrophobic balance.

## 7. Conclusions

An electroactive organic polypyrrole coating may be useful in various applications where conductive substrates are applied. The extent of usability is closely related to the structure and topographical features imposed by the synthesis conditions. Among widely used techniques, ED provides an evenly distributed coating that facilitates practical applications. The roughness of the coatings is both the result of the deposition process and the key reason for the observed characteristic features, like the effective surface area, mechanical properties, and wettability. By acknowledging the conditions that influence the synthesis process, like potential boundaries, the applied deposition method, solution composition, and utilized additives, one may prescribe in advance the proper procedure for planned application requirements. Among many important features of functional coatings, one of the most important is adhesion, as it determines the usability of the proposed systems. Several elegant strategies have been discussed that enhance the strength of bonding between polymer and substrate at the synthesis stage via either pre- or post-synthesis modifications. The film thickness controlled by the applied electrodeposition method is a parameter that can also modulate the roughness, as there is a tendency for the R_a_ value to increase depending on the extent of the surface coverage of the substrate. Inevitably, this translates immediately into other aspects, like diffusion restriction and ion-mobility issues, so several factors should be considered while planning the deposition stage. Overall, the knowledge of the roughness of the coating assists markedly in evaluating the synthetic process as well as adjusting course of coating application.

## Data Availability

No new data were created or analyzed in this study. Data sharing is not applicable to this article.
